# Overexpression of RASD1 inhibits glioma cell migration/invasion and inactivates the AKT/mTOR signaling pathway

**DOI:** 10.1038/s41598-017-03612-0

**Published:** 2017-06-09

**Authors:** Shangfeng Gao, Lei Jin, Guangping Liu, Peng Wang, Zonghan Sun, Yujia Cao, Hengliang Shi, Xuejiao Liu, Qiong Shi, Xiuping Zhou, Rutong Yu

**Affiliations:** 1 Institute of Nervous System Diseases, Xuzhou Medical University, 84 West Huai-Hai Road, Xuzhou, 221002 Jiangsu China; 2 Brain Hospital, Affiliated Hospital of Xuzhou Medical University, 99 West Huai-Hai Road, Xuzhou, 221002 Jiangsu China; 3 The Graduate School, Xuzhou Medical University, 209 Tong-Shan Road, Xuzhou, 221004 Jiangsu China

## Abstract

The RAS signaling pathway is hyperactive in malignant glioma due to overexpression and/or increased activity. A previous study identified that *RASD1*, a member of the RAS superfamily of small G-proteins, is a significantly dysregulated gene in oligodendroglial tumors that responded to chemotherapy. However, the role and mechanism of RASD1 in the progression of human glioma remain largely unknown. In the present study, by analyzing a public genomics database, we found that high levels of RASD1 predicted good survival of astrocytoma patients. We thus established lentivirus-mediated RASD1-overexpressing glioma cells and found that overexpressing RASD1 had no significant effects on glioma cell proliferation. However, the overexpression of RASD1 inhibited glioma cell migration and invasion. In the intracranial glioma xenograft model, the overexpression of RASD1 significantly reduced the number of tumor cells invading into the surrounding tissues without affecting the tumor size. An intracellular signaling array revealed that the phosphorylation of both AKT and the S6 ribosomal protein significantly decreased with RASD1 overexpression in glioma cells. Interestingly, RASD1 protein levels were significantly higher in grade II and grade III astrocytoma tissues than in nontumorous brain tissues. These findings suggest that the upregulation of RASD1 in glioma tissues may play an inhibitory role in tumor expansion, possibly through inactivating the AKT/mTOR signaling pathway.

## Introduction

Glioma is the most common and aggressive primary brain tumor. Despite the advances in surgery and adjuvant therapy, the median survival of glioblastoma patients is only 1 year^1, 2^. The highly migratory and invasive nature of glioma promotes the infiltration of these tumors into normal brain parenchyma, thereby complicating surgical resection and field radiotherapy. Many genes are dysregulated in gliomas, and aberrant overexpression and/or increased activity of RAS proteins occur in most adult gliomas, making them important targets for brain glioma therapy^3–5^.

RASD1, also named Dexras1 or AGS1, is a member of the RAS superfamily of small G-proteins^6^, which plays important roles in tumor growth and expansion. It was first discovered as a dexamethasone-inducible cDNA in mouse corticotroph cells (hence the name Dexras1)^7^ and subsequently as a receptor-independent activator of heterotrimeric G-protein signaling (hence the name AGS1)^8^. Human RASD1 is widely expressed in a variety of tissues, including the brain, heart, liver, lung, kidney and pancreas^6^.

The *RASD1* gene maps to human chromosome 17p11.2, a region associated with a high incidence of loss of heterozygosity and deletions in cancers^9, 10^. Vaidyanathan *et al*. reported that the transfection of the *RASD1* gene into the human lung adenocarcinoma cells markedly inhibited the cell growth and induced cell apoptosis^11^. Moreover, a similar suppression of cell growth by RASD1 was also observed in breast cancer cells^11^. Subsequent studies have shown that the loss of RASD1 contributes to the insensitivity to antigrowth signals in renal cell carcinoma^12^ and that B-cell proliferation and activity are negatively regulated by RASD1^13^. These findings indicate that RASD1, a member of the RAS superfamily of small G-proteins that often promotes cell growth and tumor expansion^3–5^, may play an active role in preventing aberrant cell growth.

**Figure 1:**
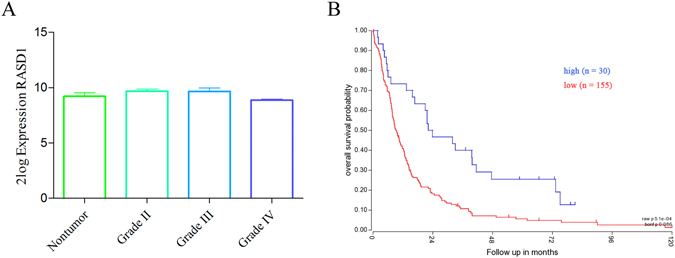
RASD1 gene expression and prognosis analysis in astrocytoma tissues. Patient data and gene expression datasets were obtained from R2 genomics database. (**A**) No significant differences were found in the RASD1 levels between the nontumor group (n = 8) and grade II (n = 13) or grade III (n = 16) or grade IV (n = 159) astrocytoma group. (**B**) High RASD1 levels were associated with good survival in astrocytoma patients (n = 185, *P* = 0.086).

In brain glioma, one study identified *RASD1* as a significantly dysregulated gene in oligodendroglial tumors that responded to chemotherapy^14^. We thus analyzed the R2 genomics database (http://hgserver1.amc.nl/cgi-bin/r2/main.cgi). However, *RASD1* gene expression showed no significant changes in grade II (n = 13), grade III (n = 16) or grade IV (n = 159) astrocytoma tissues compared to the nontumor group (n = 8) (all *P* > 0.05, Fig. 1A). Interestingly, higher RASD1 levels predicted better survival in astrocytoma patients (n = 185, *P* = 0.086) (Fig. 1B). These clinical data suggested the potential involvement of RASD1 in the progression of human glioma.

In the present study, we first established stable glioma cells by overexpressing RASD1 lentivirus plasmid and then investigated the effects of overexpressing RASD1 on the proliferation, migration, and invasion of glioma cells. Secondly, we screened the glioma cells to reveal the changes in some important signaling pathways by RASD1 overexpression. Thirdly, we set up an intracranial glioma xenograft model in nude mice to observe the effects of RASD1 overexpression on tumor growth and expansion *in vivo*. Lastly, we assessed the expression of RASD1 protein levels in various grades of astrocytoma tissues.

## Results

### Efficiency of RASD1 overexpression in glioma cells

Fluorescence microscopy observations revealed that 90% of lentivirus-infected U251 cells and U87 cells had GFP fluorescence (sFig. 1A). Western blot further confirmed that exogenous RASD1 was abundantly overexpressed in U251 and U87 cells (sFig. 1B). In addition, we observed the efficiency of RASD1 overexpression by immunofluorescence (sFig. 2). These experiments indicated that lentivirus-mediated stable cell lines with RASD1 overexpression were successfully established.

### Overexpression of RASD1 does not affect glioma cell proliferation and cell cycle progression

**Figure 2:**
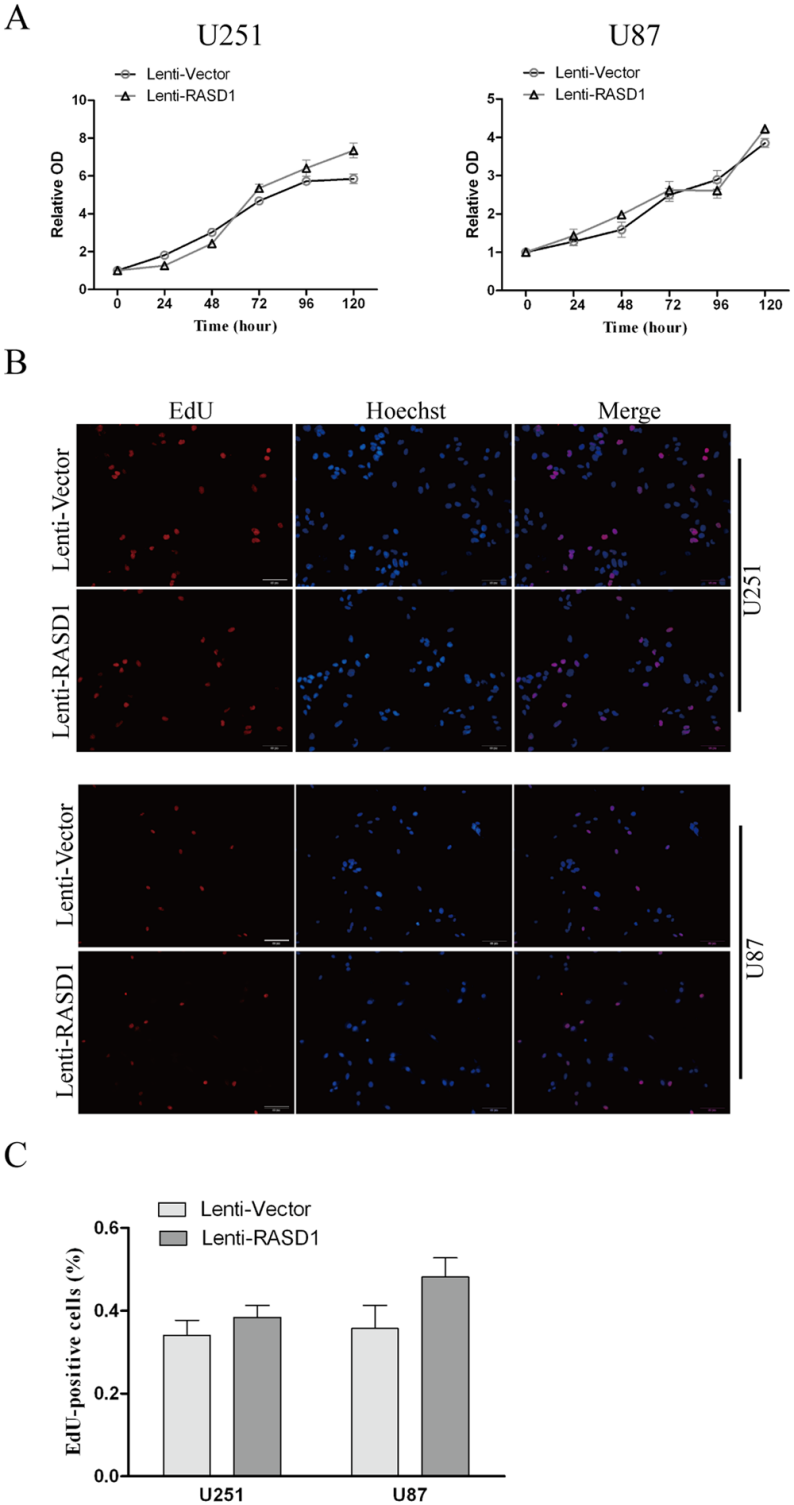
CCK-8 and EdU assays showed the effects of RASD1 overexpression on the proliferation of glioma cells. (**A**) CCK8 assay was used to detect the cell viability in U251 and U87 cells. No significant difference was found between Lenti-Vector and Lenti-RASD1 groups at any time point indicated. (**B**,**C**) EdU assay was used to evaluate the proliferation in U251 and U87 cells. Representative images for EdU-positive cells (red) and Hoechst-stained nuclei (blue) are shown in (**B**). Scale bars: 50 μm. Quantification of the percentage of EdU-positive cells is shown in (**C**). There was no significant difference in the percentage of EdU-positive cells between Lenti-Vector and Lenti-RASD1 groups.

**Figure 3:**
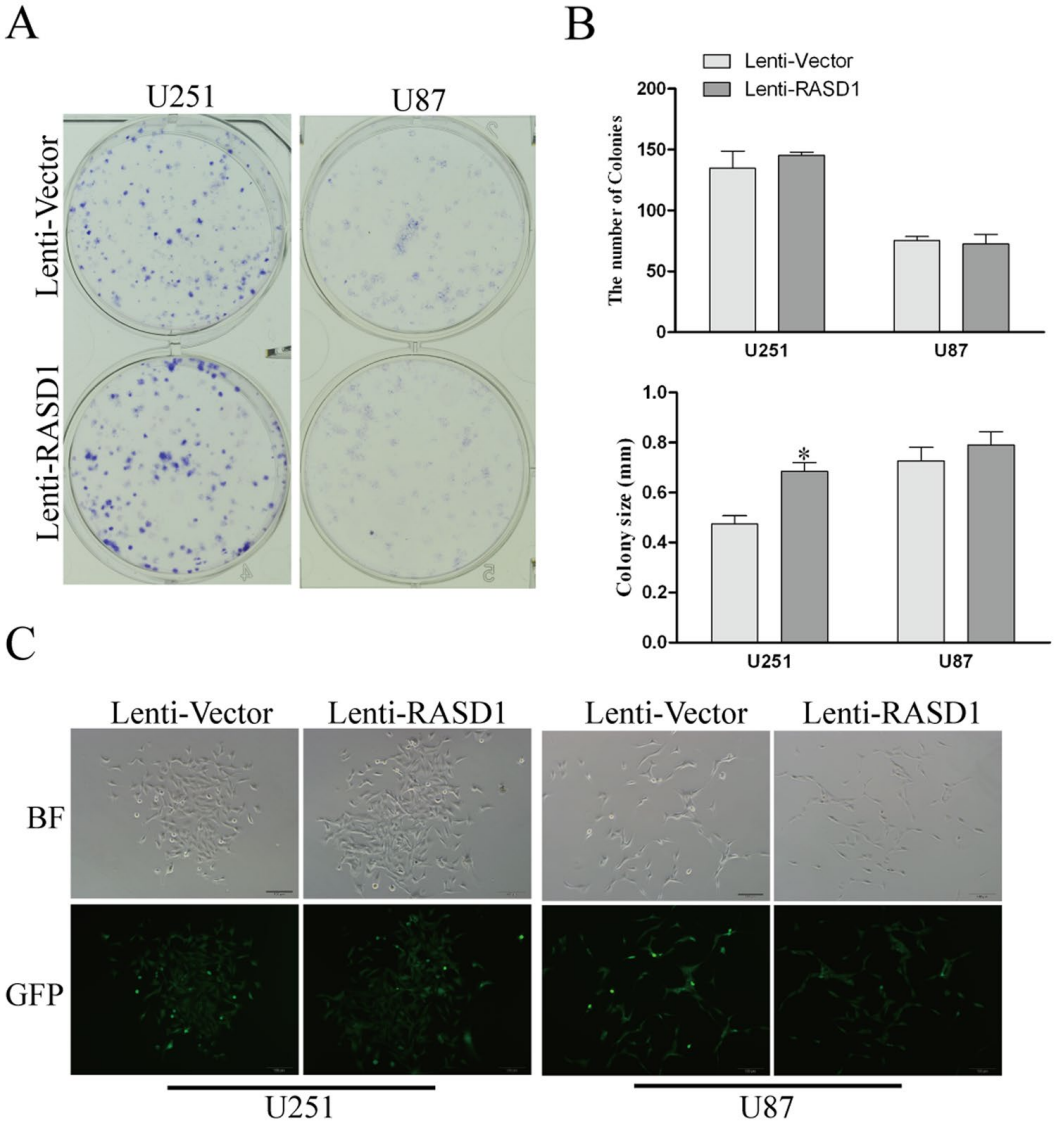
Colony formation assay showed the effects of RASD1 overexpression on the proliferation of glioma cells. Colony formation assay was used to assess the proliferation in U251 and U87 cells. Representative images for plate colony and single cell colony are shown in (**A** and **C**), respectively. Scale bar: 100 μm. Quantification for the number and size of colonies are shown in (**B**). There was no significant difference in the number of colonies between Lenti-Vector and Lenti-RASD1 groups. The colony size was much larger in Lenti-RASD1 group than in Lenti-Vector group in U251 cells, but not in U87 cells. *P < 0.05.

**Figure 4:**
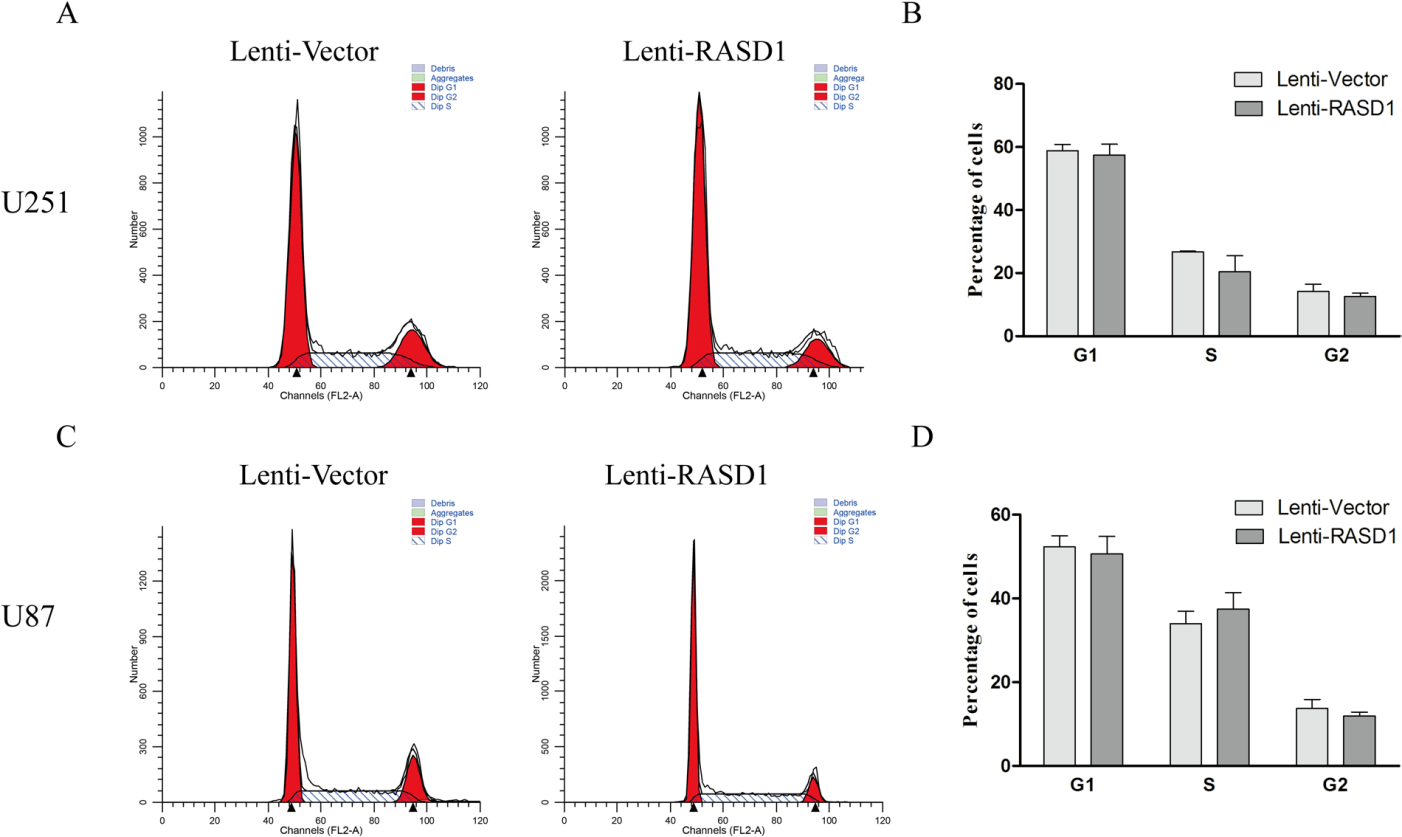
Flow cytometry exhibited the effects of RASD1 overexpression on the cell cycle progression in glioma cells. Flow cytometry was employed to analyze the cell cycle progression in Lenti-Vector and Lenti-RASD1 cells. Representative histograms for U251 and U87 cells are shown in (**A** and **C**), respectively. Quantification of the percentage of cells is shown in (**B**) (U251) and (**D**) (U87). Overexpressing RASD1 had no significant effects on the percentage of cells at any indicated stages of cell cycle.

CCK8 assays showed that there was no significant difference in the cell viability between Lenti-Vector and Lenti-RASD1 groups either in U251 cells or in U87 cells at any time point indicated (all *P* > 0.05, Fig. 2A). Additionally, the number of EdU-positive cells showed no significant differences in either U251 (*P* = 0.376) or U87 (*P* = 0.138) cells (Fig. 2B,C). Furthermore, colony formation assays revealed that the overexpression of RASD1 had no significant effects on the number of colonies (*P* = 0.513 for U251 and *P* = 0.763 for U87) (Fig. 3A,B). However, overexpressing RASD1 increased the size of colonies in U251 cells (*P* < 0.01), but did not significantly affect the size of colonies in U87 cells (*P* = 0.432) (Fig. 3). Flow cytometry showed that the percentage of cells in G1, S and G2 showed no significant changes between the Lenti-Vector and Lenti-RASD1 groups in either U251 cells (*P* ≥ 0.280, Fig. 4A,B) or U87 cells (*P* ≥ 0.385, Fig. 4C,D). These data suggested that the overexpression of RASD1 had no remarkable effects on proliferation and cell cycle progression in glioma cells.

### The overexpression of RASD1 results in cytoskeleton rearrangement and a reduction in glioma cell migration and invasion

**Figure 5:**
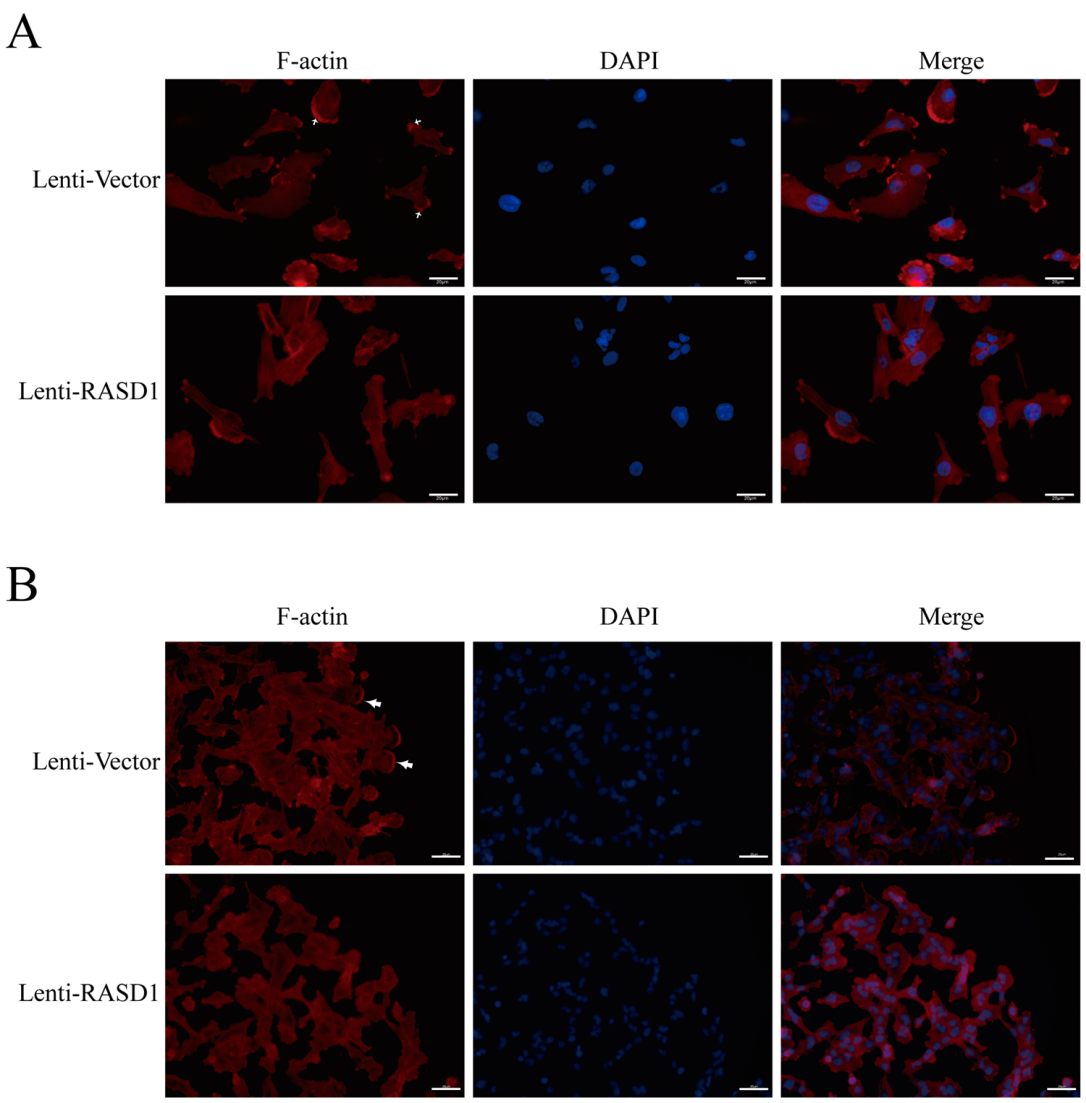
Overexpression of RASD1 leads to cytoskeleton rearrangement in glioma cells. Phalloidin staining was used to observe the F-actin cytoskeleton in Lenti-Vector and Lenti-RASD1 U251 cells. At the resting state, overexpression of RASD1 resulted in less aggregation of actin cytoskeleton on the cell membrane (arrows in **A**). During the process of cell migration induced by wound injury, Lenti-Vector cells had more actin-driven protrusions at the leading edge of cell motility than Lenti-Vector cells (arrows in **B**). Scale bars: 20 μm.

Phalloidin staining showed that F-actin in the Lenti-Vector U251 cells aggregated in the cell membrane and was less distributed in the cytoplasm, while F-actin in the Lenti-RASD1 cells was diffusely distributed in the cytoplasm (Fig. 5A). During the process of cell migration following wound injury, actin-driven protrusions were concentrated at the leading edge of cell motility in the Lenti-Vector group, whereas F-actin was still diffusely distributed in the Lenti-RASD1 group (Fig. 5B).

**Figure 6:**
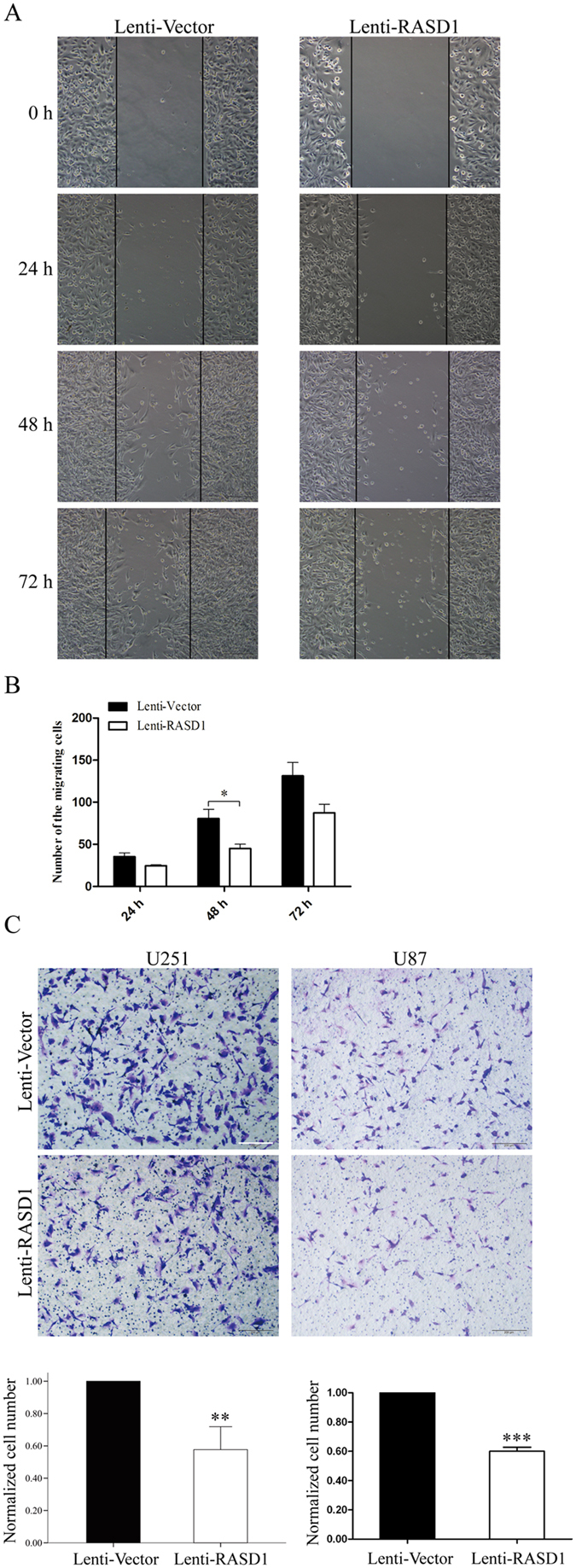
Overexpression of RASD1 inhibits the migration ability of glioma cells. (**A**,**B**) A wound healing assay was used to evaluate the migration ability in U251 cells. Overexpression of RASD1 significantly decreased the number of cells that migrated to the central area at 48 h after wound injury. (**C**) A transwell migration assay was performed in U251 and U87 cells. Overexpression of RASD1 significantly decreased the number of cells that migrated to the chamber. Scale bar: 100 μm; *P < 0.05; **P < 0.01; **P < 0.001.

Actin cytoskeleton rearrangement has been characterized to contribute to cell movement^15^. We therefore used wound healing assays to observe the effects of overexpressing RASD1 on glioma cell migration. Compared with the Lenti-Vector U251 cells, the number of cells that migrated to the central area at 24 h (*P* = 0.075), 48 h (*P* = 0.042) and 72 h (*P* = 0.081) after wound injury was remarkably decreased in Lenti-RASD1 cells (Fig. 6A,B). Furthermore, transwell migration assays showed that overexpression of RASD1 significantly decreased the number of cells that migrated to the chamber in both U251 cells and U87 cells (*P* = 0.009 and *P* < 0.001, respectively, Fig. 6C).

**Figure 7:**
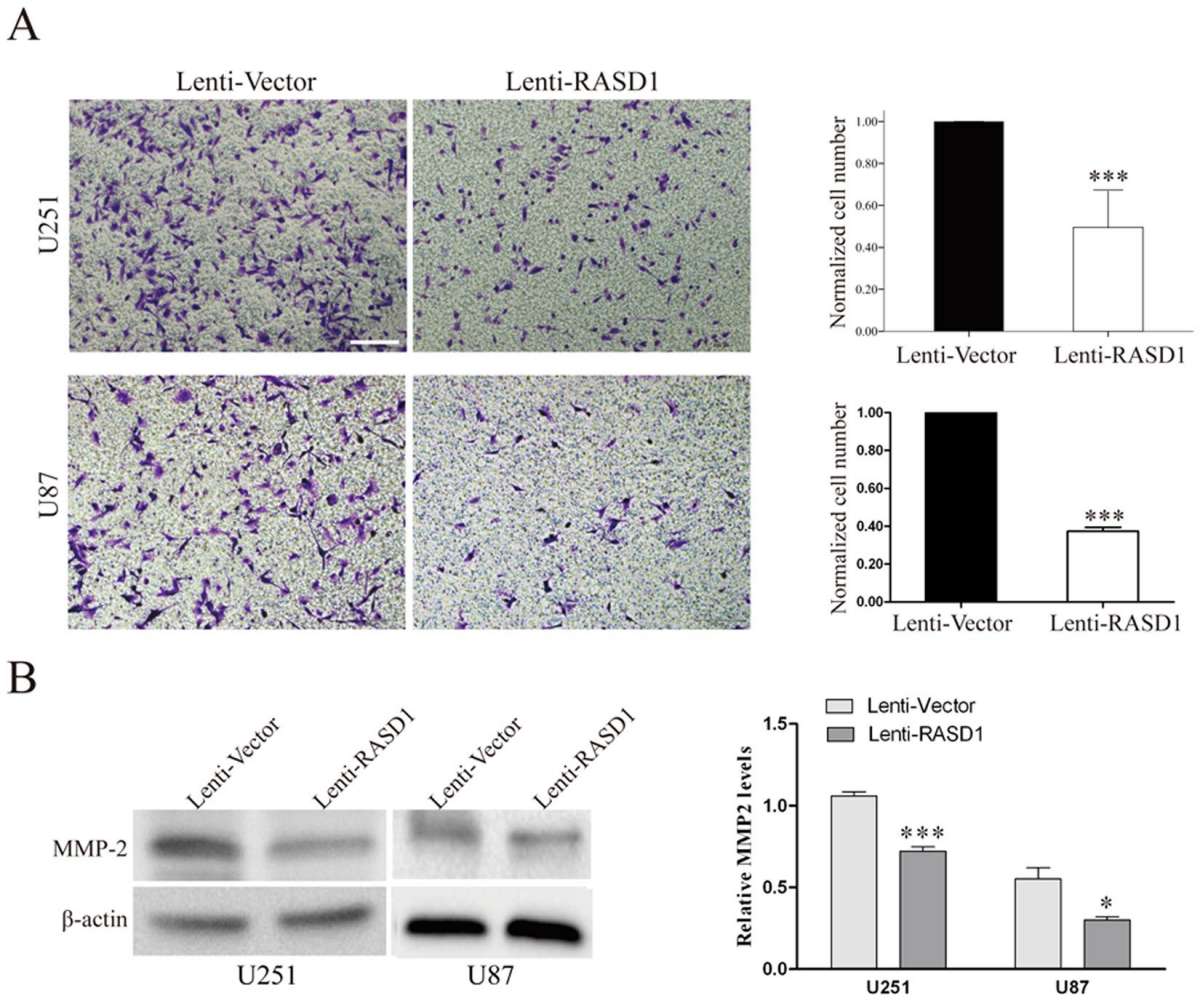
Overexpression of RASD1 inhibits the invasion ability of glioma cells. (**A**) A transwell invasion assay was performed in U251 and U87 cells. Overexpression of RASD1 significantly decreased the number of cells that passed through the Matrigel. Scale bar: 100 μm; ***P < 0.001. (**B**) MMP2 Western blot analysis was employed to reflect the invasion ability of U251 and U87 cells. Overexpression of RASD1 significantly decreased the protein levels of MMP2. *P < 0.05; ***P < 0.001.

We used the transwell assay in the presence of Matrigel to observe the effects of RASD1 overexpression on glioma cell invasion. The overexpression of RASD1 significantly reduced the number of cells that passed through the Matrigel in both U251 cells and U87 cells (all *P* < 0.001, Fig. 7A). MMP2 is a critical enzyme for the degradation of the extracellular matrix and thus contributes to glioma cell migration and invasion^16^. We found that overexpressing RASD1 caused significant decreases in the MMP2 protein levels in both U251 cells and U87 cells (*P* < 0.001 and *P* = 0.023, respectively, Fig. 7B). Taken together, these findings suggested that the overexpression of RASD1 inhibited glioma cell migration and invasion.

### The overexpression of RASD1 results in the inactivation of the AKT/mTOR pathway in glioma cells

**Figure 8:**
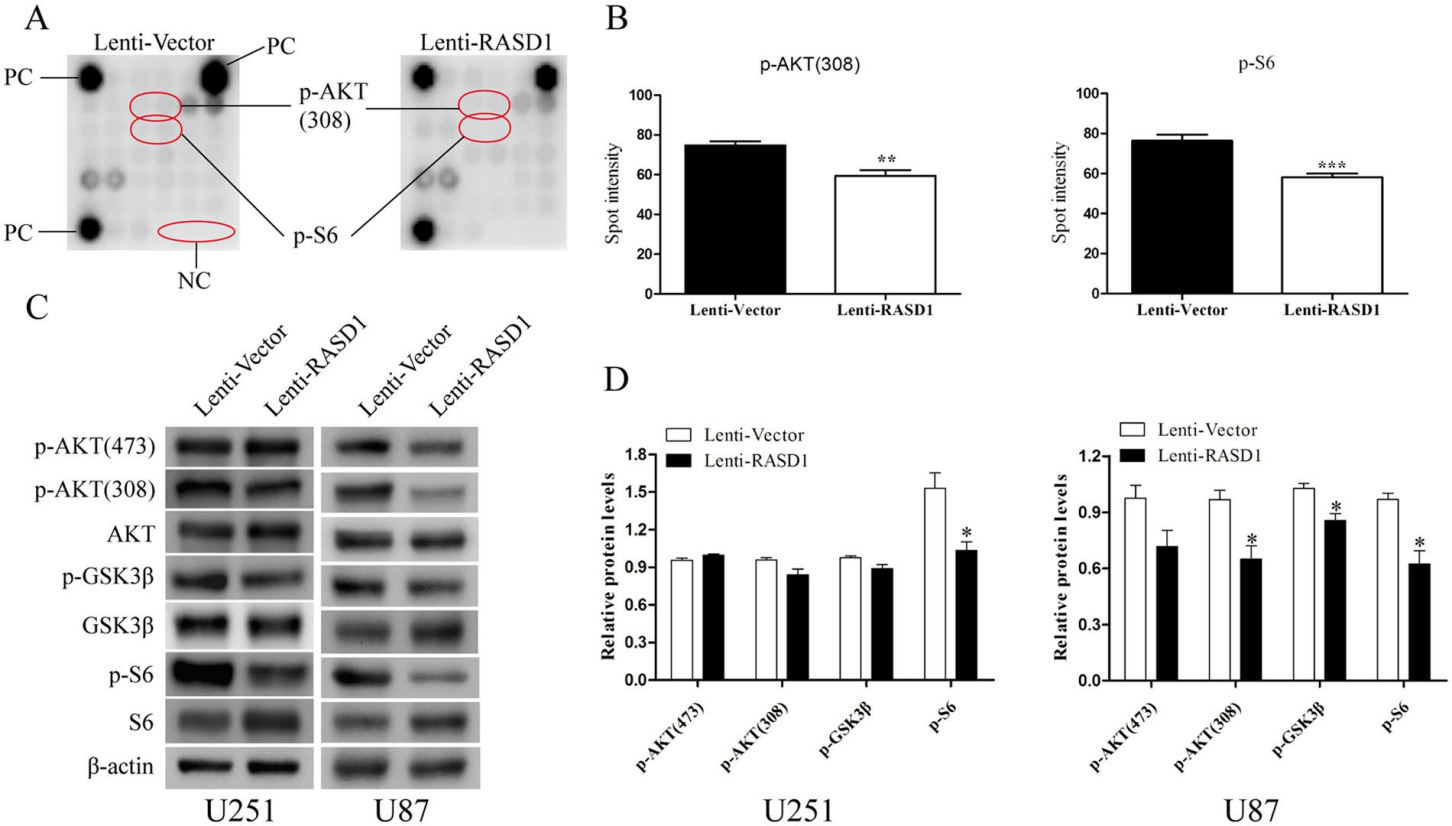
Overexpression of RASD1 suppresses AKT/mTOR signaling in glioma cells. (**A**,**B**) Antibody array was used to screen important signaling molecules in Lenti-RASD1 and Lenti-Vector U251 cells. Representative spot pictures are shown in (**A**), quantification for the spot intensity of those molecules with significant changes, i.e., p-AKT (308) and p-S6, is shown in (**B**). **P < 0.01; ***P < 0.001. (**C**,**D**) Western blot was used to confirm the antibody array data in U251 and U87 cells. Overexpression of RASD1 showed a significant reduction of p-S6 levels in U251 cells and significant decreases of p-AKT (308), p-GSK3β and p-S6 levels in U87 cells. *P < 0.05.

To explore the candidate mechanisms by which RASD1 exerted its effect on glioma cell migration and invasion, we performed an intracellular signaling array that included 18 important signaling molecules with phosphorylation or cleavage (Supplementary Table 1) in stable RASD1-overexpressing glioma cells. This array revealed that the p-AKT (Thr308) and p-S6 ribosomal protein (Ser235⁄236) levels were significantly reduced in Lenti-RASD1 cells compared to those in Lenti-Vector cells (*P* = 0.002 and *P* = 0.001, respectively, Fig. 8A,B). However, the levels of other phosphorylated or cleaved proteins, such as ERK1/2, P53 and caspase-3, were not significantly affected by RASD1 overexpression (Supplementary Table 1). As confirmed by Western blot, overexpressing RASD1 caused remarkable deceases in the levels of p-AKT (Thr308) and p-S6 in U251 cells (*P* = 0.079 and *P* = 0.024, respectively) and significant decreases in the levels of p-AKT (Thr308) (*P* = 0.021), p-GSK3β (*P* = 0.019) and p-S6 (*P* = 0.011) in U87 cells (Fig. 8C,D). These results indicated that the overexpression of RASD1 resulted in the inactivation of the AKT/mTOR pathway in glioma cells.

### Overexpressing RASD1 suppresses tumor cell invasion in the intracranial glioma xenograft model

**Figure 9:**
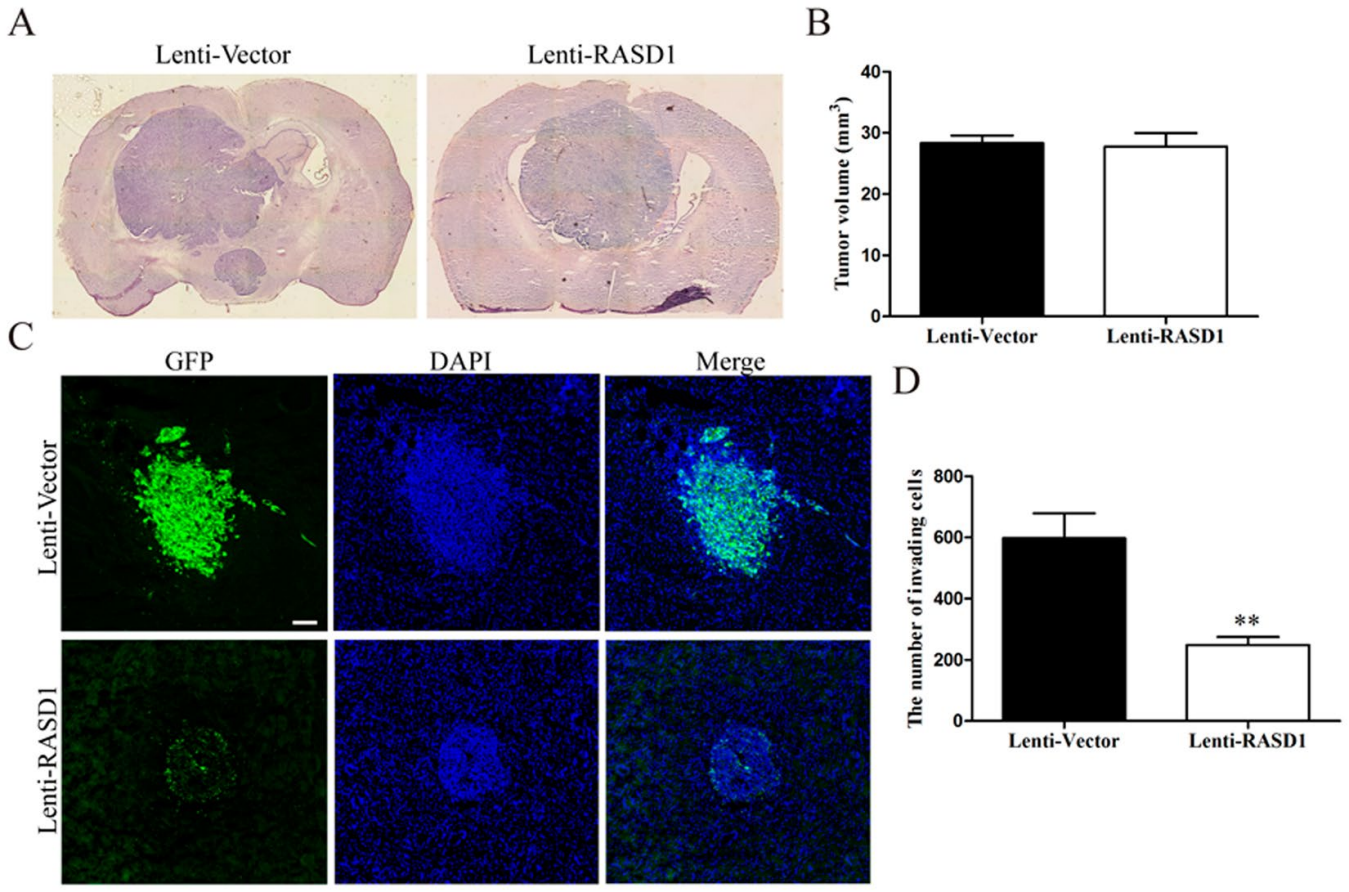
The influence of RASD1 overexpression on tumor growth and expansion in an intracranial glioma model. (**A**,**B**) An intracranial glioma model was established in nude mice, and hematoxylin and eosin (H&E) staining was performed to evaluate the tumor growth in the coronal sections. Representative H&E images from Lenti-Vector and Lenti-RASD1 groups are shown in (**A**), and the quantification graph for tumor size is shown in (**B**). Overexpressing RASD1 had no significant effects on the tumor volume (n = 3 per group). (**C**,**D**) Fluorescence micrograph of mouse brain section obtained 2 weeks after transplantation of glioma cells into the right striatum of nude mice. Representative fluorescent images from Lenti-Vector and Lenti-RASD1 groups are shown in (**C**). DAPI was used to stain the nucleus. Scale bar: 50 μm. Quantification graph of the invading cell numbers is shown in (**D**) (n = 3 for each group). Overexpression of RASD1 significantly reduced the number of invading cells outside the tumor core. **P < 0.01.

An intracranial glioma xenograft model was established in nude mice to investigate the effect of RASD1 overexpression on glioma growth and expansion *in vivo*. As shown in Fig. 9A,B, the tumor volume was not significantly different between tumors derived from Lenti-Vector and Lenti-RASD1 U87 cells (*P* = 0.698). However, the GFP-labeled tumor cells invaded the brain much less extensively in the Lenti-RASD1 group than in the Lenti-Vector group, as evidenced by the decreased number of invading cells outside the tumor core in the Lenti-RASD1 group (*P* = 0.006, Fig. 9C,D). This *in vivo* evidence further supported an inhibitory effect of RASD1 overexpression on glioma cell invasion.

### The expression profile of the RASD1 protein in various grades of astrocytoma tissues

**Figure 10:**
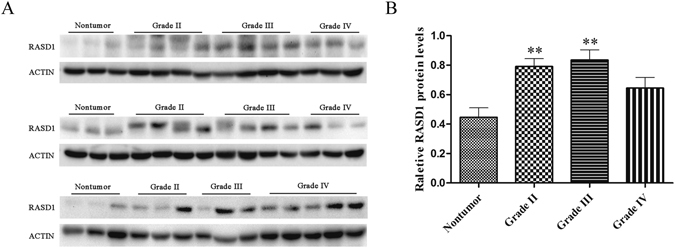
The changes of RASD1 protein levels in various grades of astrocytoma tissues. Western blot analysis was used to measure the protein levels of RASD1 in nontumor and astrocytoma tissues. Blot pictures are shown in (**A**). Quantification graphs are shown in (**B**). RASD1 protein levels were significantly increased in astrocytoma tissues of grade II (n = 11) and grade III (n = 11), but not in grade IV (n = 11), compared to the nontumor group (n = 9). **P < 0.01.

In total, 42 astrocytoma samples collected during the surgery were subjected to Western blot analysis. As shown in Fig. 10, the protein levels of RASD1 increased in grade II (*P* = 0.005, n = 11) and grade III (*P* = 0.001, n = 11) astrocytoma tissues compared to nontumor brain tissues (n = 9), with no significant changes in the grade IV glioma tissues (*P* = 0.179, n = 11). These data indicated that RASD1 protein levels changed in various pathological grades of astrocytoma tissues.

## Discussion

In the present study, we demonstrated that the overexpression of RASD1 significantly inhibited the migration and invasion of glioma cells, without affecting cell proliferation. In line with these *in vitro* results, overexpressing RASD1 markedly suppressed glioma expansion in the intracranial glioma xenograft model. In addition, a significantly positive association of RASD1 levels with the overall survival of astrocytoma patients was found by analyzing a public database. These findings indicate that targeting RASD1 is a promising therapeutic strategy for preventing tumor cell expansion in human brain glioma.

RASD1 is a member of the RAS superfamily of small GTPases^6^. As such, it is presumed to have an oncogenic function. However, the available evidence is inconsistent and does not support this presumption. RASD1 was found to be elevated in osteosarcoma^17^ and in prostate cancer^18^, and overexpressing RASD1 enhanced the proliferation of osteosarcoma cells^19^. In contrast, the overexpression of RASD1 resulted in the inhibition of growth in breast cancer, renal cell carcinoma and lung adenocarcinoma cell lines^11, 12^. In our study, overexpressing RASD1 had no significant influence on the proliferation of glioma cells, as determined by CCK8, EdU and colony formation assays. Cell cycle progression was also not affected in the Lenti-RASD1 cells. Interestingly, we found that the overexpression of RASD1 significantly inhibited both the migration and invasion abilities of glioma cells. RASD1 belongs to a distinct group of RAS-like monomeric G proteins, with ∼35% similarity to each of the major RAS subfamilies^20^. These findings suggested that RASD1, unlike other RAS family members, may play different roles in various cancer cells.

We explored the candidate mechanisms in Lenti-RASD1 glioma cells by an intracellular signaling array that can simultaneously reflect several important signaling cascades, e.g., MAPK, mTOR, and AKT. We found that the overexpression of RASD1 remarkably suppressed the phosphorylation of AKT (Thr308), GSK3β and S6 ribosome protein in glioma cells. GSK3β is a downstream target of AKT, and phosphorylation of S6 at Ser235/236 reflects mTOR activation. Thus, we demonstrated for the first time the inhibitory effects of RASD1 overexpression on the AKT/mTOR pathway, which is frequently activated in gliomagenesis^2^. Considering the close relationship between the AKT/mTOR pathway and the epithelial-mesenchymal transition^21, 22^, we speculated that RASD1 inhibits the migration and invasion of glioma cells possibly through the AKT-mediated epithelial-mesenchymal transition. This was further supported by our findings that the overexpression of RASD1 reduced the accumulation of F-actin in the lamellipodia and invasion-filopodia. Further studies are needed to uncover the specific mechanism by which RASD1 regulates AKT/mTOR signaling.

A previous study reported that ERK1/2 was weakly activated after RASD1 overexpression in COS-7 cells^23^. Additionally, we found no significant alterations of p-ERK1/2, p-JNK1/2 and p-P38 in RASD1-overexpressing glioma cells by antibody array (Supplementary Table 1), which paralleled the insignificant effects of RASD1 overexpression on glioma cell proliferation. It should be noted that there is a greater increase of light-induced p-ERK1/2 expression in the suprachiasmatic nucleus of RASD1-deficient mice^24^. Therefore, RASD1-ERK1/2 cross-talk may depend on the cell type or the stimuli, which should be considered in future investigations.

In the R2 genomics database, there was no significant difference in *RASD1* gene expression between the nontumor and astrocytoma groups (Grade II or III or IV). However, we found a significant increase in RASD1 protein levels in astrocytoma tissues, especially pathological grade II and grade III tissues. These results indicated that the upregulation of the RASD1 protein in astrocytoma tissues might not result from the increased transcription of RASD1. Similar to other members of the RAS family, RASD1 undergoes posttranslational modifications by the farnesylation of its CAAX box^25^. The isoprenylation of this CAAX consensus site is required for the translocation of RAS proteins to the cell membrane^26^ and can affect protein stability^27^. The increased stability of farnesylated RASD1 may provide an explanation for the higher levels of RASD1 observed in astrocytoma tissues of grade II and grade III. Further investigations are warranted to confirm this supposition.

In conclusion, both *in vitro* and *in vivo* findings suggest that RASD1 may play an inhibitory role in cell migration and invasion in brain glioma, although the loss of function data are lacking due to low expression of the RASD1 protein in the glioma cell lines studied. The mechanism involves the inactivation of the AKT/mTOR pathway and cytoskeleton rearrangement. In addition, we found that high levels of RASD1 predicted good survival in astrocytoma patients. Therefore, RASD1 may serve as an attractive target for the development of novel diagnostic, prognostic and therapeutic approaches to glioma management.

## Materials and Methods

### Patient data analysis

Patient data and gene expression datasets were obtained from R2: microarray analysis and visualization platform (http://hgserver1.amc.nl/cgi-bin/r2/main.cgi). Gene expression and prognosis analyses were performed online, and all data and *P* values (log-rank test) were downloaded. All cutoff values for separating high and low expression groups were determined by the online R2 database algorithm.

### Cell lines and cell culture

The HEK293T cells and the human glioma cell lines U251 and U87 were purchased from the Shanghai Cell Bank, Type Culture Collection Committee, Chinese Academy of Sciences. The cells were grown in DMEM (293 T and U251) or MEM (U87) supplemented with 10% fetal bovine serum (Hyclone). All cell lines were cultured in a cell incubator containing 5% CO_2_ under saturated humidity at 37 °C.

### Antibodies

Antibodies against RASD1 (1:1000, Abcam, Cambridge, UK), GFP (1:500, Santa Cruz Bio., Santa Cruz, CA), MMP2 (1:200, Boster, Wuhan, China), and the signaling molecules AKT, p-AKT (Thr308 and Ser473), GSK3β, p-GSK3β, S6 and p-S6 (1:1000, Cell Signaling Technology, Denver, CO) are commercially available.

### Lentivirus construction, production and infection

The RASD1 lentivirus plasmid was constructed, produced and used to infect the glioma cells as described in our previous publication^28, 29^. Forty-eight hours after infection, the virus-infected cells were cultured in the medium containing 2.5 μg/mL puromycin (Sigma, St. Louis, MO) for selection. The surviving cells were used for the subsequent experiments. RASD1-overexpressing cells were named Lenti-RASD1; the corresponding control cells were named Lenti-Vector.

### Immunofluorescence analysis

Cells were grown on 13-mm-diameter coverslips for 2 days and then fixed with 4% paraformaldehyde and permeabilized in 0.2% Triton X-100. After being blocked in 10% normal goat serum for 30 min, the cells were incubated with rabbit anti-RASD1 antibody (Abcam, 1:200) in a humidified chamber overnight at 4 °C. On the following day, the Alexa Fluor 594-conjugated goat anti-rabbit IgG (1:200, Jackson lab) was added and incubated for 2–3 h at room temperature. F-actin was stained using 200 μL of 100 nM rhodamine-phalloidin according to the manufacturer’s protocol (Cytoskeleton, Denver, CO). The nuclei were stained with 4,6-diamidino-2-phenylindole (DAPI; 1:1000; Sigma). The cells were observed and recorded using a fluorescence microscope.

### Cell growth assay

Cell viability was measured with a Cell Counting Kit-8 (CCK-8, Dojindo, Japan). The single cell suspension (5 × 10^4^/mL, 100 μL) was placed in a 96-well plate and cultured for 6 h, 12 h, 24 h, 48 h, 72 h, 96 h and 120 h. Then, 10 μL of the CCK-8 reagent was added to each well and incubated for another 1.5 h. Then, the absorbance (value) at 450 nm was measured using a scanning microplate reader. Cell viabilities at individual time point were normalized to those at 6 h.

### EdU assay

The 5-ethynyl-20-deoxyuridine (EdU) incorporation assay was performed with an EdU assay kit (Ribobio, Guangzhou, China) according to the manufacturer’s instructions. Cells were seeded into 96-well plates at 5 × 10^3^ cells per well for 24–48 h and then exposed to 50 μM EdU for 4 h at 37 °C. Subsequently, the cells were fixed with 4% paraformaldehyde and then permeabilized with 0.5% Triton X-100. Finally, the cells reacted with 100 μL of 1 × Apollo® reaction cocktail for 30 min, followed by incubation with 100 μL of Hoechst 33342 (5 μg/mL). The images were taken with an Olympus IX-71 inverted microscope (Tokyo, Japan). The percentage of EdU-positive cells was calculated by dividing the number of EdU-positive cells by the number of Hoechst-stained cells.

### Colony formation assay

Two hundred cells were seeded in a 6-cm dish and cultured for 2–3 weeks. The cells were fixed with methanol and stained with 0.05% crystal violet to assess colony staining. After being washed with PBS, the plates were photographed with a camera. Colonies containing more than 50 cells were counted. The colony size was analyzed by Image J software (National Institutes of Health, Bethesda, MD).

### Flow cytometry

The cell cycle was assessed by flow cytometry with a commercial kit (NewMed Cytomics, Suzhou, China) according to the manufacturer’s protocol. Briefly, cells were trypsinized into a single cell suspension and collected by centrifuging at 1500 rpm. After washing with PBS two times, reagents A, B, and C from the kit were successively added into the cells. The cell suspension was filtered by a 50-mm nylon mesh and immediately analyzed by flow cytometry (BD, Franklin Lakes, NJ).

### Wound healing assay

The migration behavior of cells was evaluated using the wound healing assay. Monolayers were wounded with a plastic pipette tip, rinsed twice with PBS to remove dead cells and incubated in serum-free media. At the designated time (0 h, 24 h, 48 h, and 72 h), three randomly selected fields at the lesion border were acquired under an Olympus IX-71 inverted microscope. The number of migrating cells across the wound was counted on the images.

### Transwell invasion and migration assays

Cell invasion and migration assays were performed using a transwell system that incorporated a polycarbonate filter membrane with a diameter of 6.5 mm and pore size of 8 μm (Corning, NY) according to the manufacturer’s protocol. To assess invasion ability, filters were precoated with 10 μg of Matrigel (BD). The cell suspension (1 × 10^4^) in serum-free culture media was added to the inserts, and each insert was placed in the well of a plate filled with culture media containing 10% FBS as a chemoattractant. After 24 h of incubation at 37 °C, the noninvasive cells were removed from the upper chamber by wiping with cotton-tipped swabs, and filters were fixed with 4% paraformaldehyde for 30 min and stained with a 0.1% crystal violet solution for 30 min at room temperature. Five fields of adherent cells in each well were randomly photographed under an Olympus IX71 inverted microscope. The same experimental design was used for migration experiments, except the filters were not precoated with Matrigel. The number of cells migrating across the filters was counted on the captured images. The number of cells in the Lenti-RASD1 group was calculated by normalizing them to those in the Lenti-Vector group.

### Antibody array

Analysis of an Intracellular Signaling Array was performed according to the protocol provided by the manufacturer (Cell Signaling Technology). In brief, Lenti-Vector and Lenti-RASD1 glioma cells were lysed in lysis buffer containing 1 mM phenylmethylsulfonyl fluoride. In total, 60 μg of lysate was added to a slide coated with target-specific capture antibodies. The slide was then incubated with a biotinylated antibody cocktail. Streptavidin-conjugated horseradish peroxidase and LumiGLO reagent were used to visualize the bound detection antibody by chemiluminescence. Phosphorylation or cleavage of important signaling molecules were quantified with Image J software (National Institutes of Health).

### Glioma xenograft model and histopathology

Male BALB/c nude mice aged 5 to 6 weeks were obtained from the Experimental Animal Center of Xuzhou Medical University and housed in Individually Ventilated Caging (IVC) systems in a 12-h light/dark cycle. Lenti-RASD1 or Lenti-Vector U87 cells (1 × 10^6^) were stereotactically injected into the right striatum of nude mice as described in our previous studies^30, 31^. The mice were sacrificed when cachexia occurred in the tumor growth assay or at two weeks after glioma cell transplantation for the tumor invasion assay. The whole brains were harvested, fixed and dehydrated sequentially in 20% and 30% sucrose at 4 °C until they sank. The fresh-frozen brain was serially cut at thicknesses of 12 μm, and the section with the largest tumor area was stained with hematoxylin and eosin (H&E) or used for counting the invading cells outside the tumor core. Tumor volume was calculated according to the formula V (mm^3^) = a × b^2^/2, where a is the longest diameter, and b is the shortest diameter of the tumor. The number of invading cells outside the tumor core was quantitatively analyzed according to previous reports^32, 33^.

### Clinical samples

We collected 42 fresh specimens including 33 astrocytoma tissues (Grade II: 11, Grade III: 11, Grade IV: 11) and 9 nontumor brain tissues from the Affiliated Hospital of Xuzhou Medical University, Xuzhou, China. The nontumor brain tissues were obtained from the patients undergoing surgery for internal decompression in cerebral trauma. All astrocytoma specimens had a confirmed pathological diagnosis and were classified according to the criteria of the World Health Organization (WHO). Clinico-pathological information of all subjects was given in Supplementary Table 2.

### Protein extraction and Western blot

Total protein was extracted from the cultured cells or tissues as described previously^34^. Fifty-microgram protein samples were separated via 10% sodium dodecyl sulfate polyacrylamide gel electrophoresis (SDS-PAGE) and then electrophoretically transferred to a polyvinylidene fluoride (PVDF) membrane (Millipore, Bedford, MA). Membranes were incubated in 3% bovine serum albumin in PBS for 2 h and then treated with primary antibodies at 4 °C overnight. On the following day, membranes were incubated in horseradish peroxidase-labeled goat anti-rabbit/mouse IgG (1:4000, Pierce, Rockford, IL) and detected by an enhanced chemiluminescence detection system.

### Ethics statement

Written informed consent was obtained from each subject or legal guardian and signed by subjects and legal guardians prior to participation in the study. All experimental protocols were approved by the Medical Ethical Committee of Xuzhou Medical University, and all experimental methods were carried out in accordance with the approved guidelines of Xuzhou Medical University.

### Statistical analysis

Statistical analyses were performed using SPSS version 13.0 (SPSS Inc., Chicago, IL). The *in vitro* experiments were repeated at least three times and expressed as the means ± S.E.M. Differences in multiple groups were determined by one-way analysis of variance (ANOVA) followed by Tukey’s *post hoc* test. Comparison between two groups was performed by Student’s *t* test. *P* < 0.05 was considered to be statistically significant.

## Electronic supplementary material

Supplementary information

## References

[1] Sherman JH (2011). Neurosurgery for brain tumors: update on recent technical advances. Current neurology and neuroscience reports..

[2] Wen PY, Kesari S (2008). Malignant gliomas in adults. The New England journal of medicine..

[3] Lo HW (2010). Targeting Ras-RAF-ERK and its interactive pathways as a novel therapy for malignant gliomas. Current cancer drug targets..

[4] Shi Z (2014). MiR-124 governs glioma growth and angiogenesis and enhances chemosensitivity by targeting R-Ras and N-Ras. Neuro-oncology..

[5] Wang XR (2013). Overexpressed let-7a inhibits glioma cell malignancy by directly targeting K-ras, independently of PTEN. Neuro-oncology..

[6] Tu Y, Wu C (1999). Cloning, expression and characterization of a novel human Ras-related protein that is regulated by glucocorticoid hormone. Biochimica et biophysica acta..

[7] Kemppainen RJ, Behrend EN (1998). Dexamethasone rapidly induces a novel ras superfamily member-related gene in AtT-20 cells. The Journal of biological chemistry..

[8] Cismowski MJ (1999). Genetic screens in yeast to identify mammalian nonreceptor modulators of G-protein signaling. Nature biotechnology..

[9] Koga T, Iwasaki H, Ishiguro M, Matsuzaki A, Kikuchi M (2002). Losses in chromosomes 17, 19, and 22q in neurofibromatosis type 1 and sporadic neurofibromas: a comparative genomic hybridization analysis. Cancer genetics and cytogenetics..

[10] Stacey MW, Wang J, Byrd RL, Liu JM, Kearns WG (1999). Nuclear receptor co-repressor gene localizes to 17p11.2, a frequently deleted band in malignant disorders. Genes, chromosomes & cancer..

[11] Vaidyanathan G (2004). The Ras-related protein AGS1/RASD1 suppresses cell growth. Oncogene..

[12] Dalgin GS, Holloway DT, Liou LS, DeLisi C (2007). Identification and characterization of renal cell carcinoma gene markers. Cancer informatics..

[13] Lindsey JW (2007). Dexamethasone-induced Ras-related protein 1 is a potential regulatory protein in B lymphocytes. International immunology..

[14] Shaw EJ (2011). Gene expression in oligodendroglial tumors. Cellular oncology..

[15] Ananthakrishnan R, Ehrlicher A (2007). The forces behind cell movement. International journal of biological sciences..

[16] Konnecke H, Bechmann I (2013). The role of microglia and matrix metalloproteinases involvement in neuroinflammation and gliomas. Clinical & developmental immunology..

[17] Both J (2012). Identification of novel candidate oncogenes in chromosome region 17p11.2-p12 in human osteosarcoma. PloS one..

[18] Baniwal SK (2010). Runx2 transcriptome of prostate cancer cells: insights into invasiveness and bone metastasis. Molecular cancer..

[19] Both, J., Wu, T., Ten Asbroek, A. L., Baas, F. & Hulsebos, T. J. Oncogenic Properties of Candidate Oncogenes in Chromosome Region 17p11.2p12 in Human Osteosarcoma. *Cytogenetic and genome research*, doi:10.1159/000451046 (2016). 10.1159/000451046 27846620

[20] Cismowski MJ, Lanier SM (2005). Activation of heterotrimeric G-proteins independent of a G-protein coupled receptor and the implications for signal processing. Reviews of physiology, biochemistry and pharmacology..

[21] Iskender, B. *et al*. Inhibition of epithelial-mesenchymal transition in bladder cancer cells via modulation of mTOR signalling. *Tumour biology: the journal of the International Society for Oncodevelopmental Biology and Medicine*, doi:10.1007/s13277-015-4695-1 (2015). 10.1007/s13277-015-4695-1 26718217

[22] Gonzalez DM, Medici D (2014). Signaling mechanisms of the epithelial-mesenchymal transition. Science signaling..

[23] Graham TE, Prossnitz ER, Dorin RI (2002). Dexras1/AGS-1 inhibits signal transduction from the Gi-coupled formyl peptide receptor to Erk-1/2 MAP kinases. The Journal of biological chemistry..

[24] Cheng HY (2006). The molecular gatekeeper Dexras1 sculpts the photic responsiveness of the mammalian circadian clock. The Journal of neuroscience: the official journal of the Society for Neuroscience..

[25] Thapliyal A, Verma R, Kumar N (2014). Small G Proteins Dexras1 and RHES and Their Role in Pathophysiological Processes. International journal of cell biology..

[26] Kato K (1992). Isoprenoid addition to Ras protein is the critical modification for its membrane association and transforming activity. Proceedings of the National Academy of Sciences of the United States of America..

[27] Basso AD, Kirschmeier P, Bishop WR (2006). Lipid posttranslational modifications. Farnesyl transferase inhibitors. Journal of lipid research..

[28] Gao, S. *et al*. Low Expression of CAPON in Glioma Contributes to Cell Proliferation via the Akt Signaling Pathway. *International journal of molecular sciences*. **17**, doi:10.3390/ijms17111859 (2016). 10.3390/ijms17111859 PMC5133859 27869735

[29] Shi H (2014). CacyBP/SIP protein is important for the proliferation of human glioma cells. IUBMB life..

[30] Liu X (2016). CRM1/XPO1 is associated with clinical outcome in glioma and represents a therapeutic target by perturbing multiple core pathways. Journal of hematology & oncology..

[31] Liu X (2015). Hugl-1 inhibits glioma cell growth in intracranial model. Journal of neuro-oncology..

[32] Li H (2015). KAP regulates ROCK2 and Cdk2 in an RNA-activated glioblastoma invasion pathway. Oncogene..

[33] Li S (2017). miR-423-5p contributes to a malignant phenotype and temozolomide chemoresistance in glioblastomas. Neuro-oncology..

[34] Shen A (2008). Identification and potential role of PSD-95 in Schwann cells. Neurological sciences: official journal of the Italian Neurological Society and of the Italian Society of Clinical Neurophysiology..

