# Supplementary material for: Overexpression of RASD1 inhibits glioma cell migration/invasion and inactivates the AKT/mTOR signaling pathway
Source: Sci Rep. 2017 Jun 9;7:3202. doi: 10.1038/s41598-017-03612-0 (PMC5466601; doi:10.1038/s41598-017-03612-0)

Supplementary Information:

Overexpression of RASD1 inhibits glioma cell migration/invasion and inactivates the AKT/mTOR signaling pathway

Shangfeng Gao[1,2,*], Lei Jin[3,*], Guangping Liu[3,*], Peng Wang[3], Zonghan Sun[3], Yujia Cao[3], Hengliang Shi[1,2], Xuejiao Liu[1,2], Qiong Shi[2], Xiuping Zhou[1], Rutong Yu[1,2]

Supplementary Figures and legends:

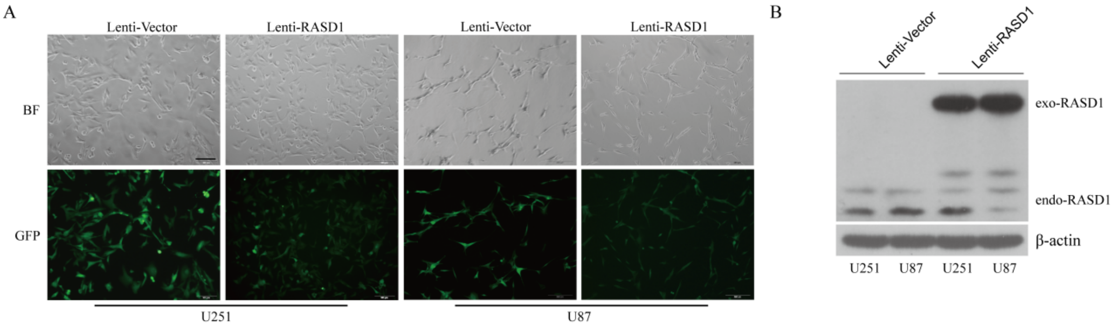

sFigure 1. Identification of the efficiency of RASD1 overexpression in glioma cells. (**A**) Combined the bright field (BF) and GFP fluorescence, 90% of glioma cells were infected by lentivirus from Vector group and RASD1 group. Scale bar: 100μm. (**B**) Western blot showed that exogenous RASD1 (exo-RASD1) was abundantly expressed in both U251 and U87 cells, while the endogenous RASD1 (endo-RASD1) was relative low. β-actin was used as a loading control.

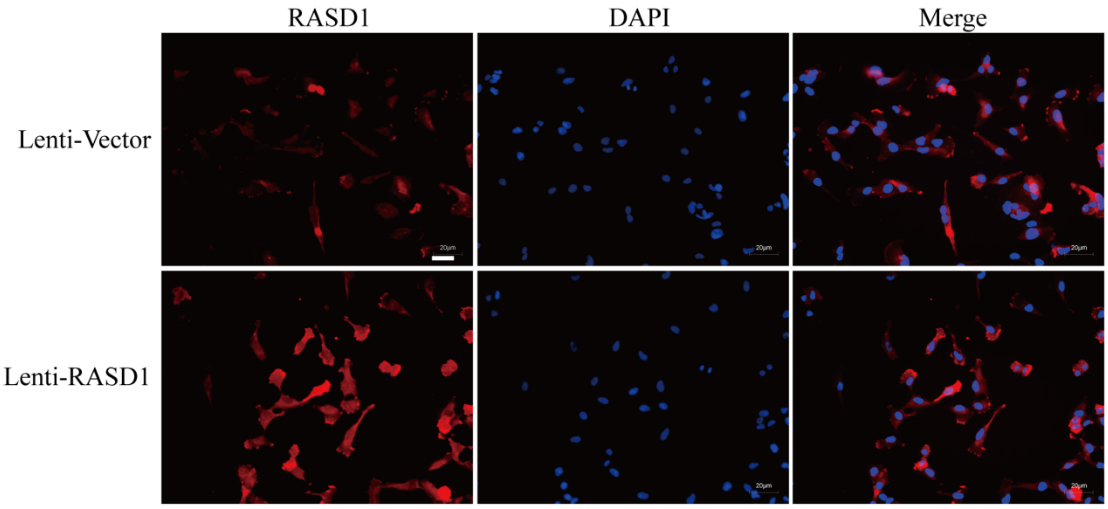

sFigure 2. Immunofluorescence observed the overexpression efficiency of RASD1 in U251 cells. Immunofluorescence was used to assess the overexpression efficiency of RASD1 in U251 cells. RASD1 expression, as reflected by the fluorescence intensity, was much higher in the Lenti-RASD1 cells than in the Lenti-Vector cells. Scale bar: 20μm

Supplementary Table 1. Summary for the antibody array

No.	Target	Site	Spot intensity in the Lenti-Vector group (Mean ± SEM)	Spot intensity in the Lenti-RASD1 group (Mean ± SEM)	*P* value (t test)	*P* value summary (t test)
3	Phospho-ERK1/2	Thr202/Tyr204	70.815 ± 2.0625 N=4	68.055 ± 4.0545 N=4	0.6808	ns
4	Phospho-Stat1	Tyr701	81.495 ± 3.69 N=4	68.745 ± 5.841 N=4	0.1145	ns
5	Phospho-Stat3	Tyr705	76.155 ± 6.882 N=4	72.03 ± 6.165 N=4	0.6709	ns
6	Phospho-Akt	Thr308	74.77 ± 1.161 N=4	59.43 ± 1.6744 N=4	0.0020	**
7	Phospho-Akt	Ser473	150.15 ± 22.395 N=4	132.195 ± 8.916 N=4	0.4834	ns
8	Phospho-AMPKα	Thr172	84.09 ± 12.105 N=4	65.505 ± 1.8495 N=4	0.1797	ns
9	Phospho-S6 Ribosomal Protein	Ser235/236	76.37 ± 1.798 N=4	58.23 ± 0.9939 N=4	0.0010	***
10	Phospho-mTOR	Ser2448	67.59 ± 3.7035 N=4	65.745 ± 2.853 N=4	0.7070	ns
11	Phospho-HSP27	Ser78	66.315 ± 5.0535 N=4	63.81 ± 2.4525 N=4	0.6718	ns
12	Phospho-Bad	Ser112	78.195 ± 3.4725 N=4	78.87 ± 1.68 N=4	0.8644	ns
13	Phospho-p70 S6 Kinase	Thr389	61.35 ± 1.9635 N=4	60.57 ± 2.7225 N=4	0.8237	ns
14	Phospho-PRAS40	Thr246	168.15 ± 20.40 N=4	137.445 ± 5.76 N=4	0.1971	ns
15	Phospho-p53	Ser15	69.9 ± 2.859 N=4	65.19 ± 1.4183 N=4	0.1897	ns
16	Phospho-p38	Thr180/Tyr182	61.59 ± 1.569 N=4	60.87 ± 2.028 N=4	0.7887	ns
17	Phospho-SAPK/JNK	Thr183/Tyr185	86.97 ± 11.6835 N=4	81.495 ± 6.2055 N=4	0.6937	ns
18	Cleaved PARP	Asp214	55.065 ± 1.4946N=4	57.69 ± 0.9554 N=4	0.1894	ns
19	Cleaved Caspase-3	Asp175	58.245 ± 1.7745 N=4	60.255 ± 1.6665 N=4	0.4429	ns
20	Phospho-GSK-3β	Ser9	81.84 ± 6.915 N=4	73.965 ± 4.0785 N=4	0.3645	ns

Note: ** $P < 0.01$; *** $P < 0.001$; ns: no significant differences

Supplementary Table 2. Clinico-pathological information for the studied subjects

Case No.	Code No.	Gender	Age (years)	Location	WHO Grade
1	919616	M	57	Cerebelli right	Nontumor
2	912226	F	54	Temporal lobe right	Nontumor
3	972078	F	49	Not available	Nontumor
4	968605	F	69	Not available	Nontumor
5	981488	M	41	Cerebelli	Nontumor
6	1095392	M	32	Not available	Nontumor
7	1004728	M	63	Frontal lobe right	Nontumor
8	941814	M	20	Cerebelli	Nontumor
9	928412	M	48	Not available	Nontumor
10	970570	M	52	Frontal lobe right	Grade II
11	1157139	M	42	Frontal lobe right	Grade II
12	1145933	F	48	Frontal lobe right	Grade II
13	1140811	F	49	Frontal lobe right	Grade II
14	1164493	M	64	Insular lobe left	Grade II
15	1190502	M	31	Frontal lobe left	Grade II
16	1158620	M	43	Temporal lobe right	Grade II
17	1196273	M	40	Frontal lobe left	Grade II
18	1152968	F	63	Temporal lobe left	Grade II
19	1110685	F	27	Frontal lobe righ	Grade II
20	1084447	F	52	Not available	Grade II
21	999737	M	52	Frontal-temporal lobe left	Grade III
22	920498	M	50	Temporal-parieto lobe right	Grade III
23	926714	M	56	Bilateral temporal lobe	Grade III
24	1164248	F	66	Frontal lobe left	Grade III
25	1191197	M	68	Parietal-occipito lobe left	Grade III
26	922050	F	19	Frontal lobe right	Grade III
27	1117547	M	47	Temporal lobe right	Grade III
28	1081283	F	58	Temporal lobe left	Grade III
29	947804	F	66	Frontal-temporal-parieto lobe left	Grade III
30	1145935	M	23	Cervical cord	Grade III
31	1029589	M	31	Frontal-temporal lobe right	Grade III
32	1147279	F	64	Cerebelli	Grade IV
33	1147166	M	58	Frontal-temporal lobe left	Grade IV
34	1141904	M	47	Temporal lobe right	Grade IV
35	1132842	F	62	Temporal lobe left	Grade IV
36	1119597	F	50	Frontal lobe right	Grade IV
37	1096129	M	61	Temporal lobe left	Grade IV
38	1077922	M	43	Temporal lobe left	Grade IV
39	1140776	M	26	Parietal-occipito lobe right	Grade IV
40	1088070	F	58	Temporal lobe right	Grade IV
41	1164493	M	66	Insular lobe left	Grade IV
42	1184604	F	34	Temporal lobe right	Grade IV

Note: F, Female; M, Male. WHO, World Health Organization.

Raw data:

rTable 1. CCK8 assay showed the cell viability in glioma cells

	Lenti-Vector				Lenti-RASD1			
0h	1	1	1	1	1	1	1	1
24h	1.796102	1.740854	1.879736	1.848754	1.25567	1.247036	1.291339	1.257143
48h	3.250375	3.03811	3.235585	2.546263	1.954639	2.496047	2.505906	2.744762
72h	4.556222	4.618902	4.634267	4.912811	4.773196	5.365613	5.529528	5.761905
96h	5.412294	5.477134	5.805601	6.186833	5.107216	6.814229	6.958662	6.769524
120h	6.086807	5.855945	6.30939	5.139858	6.183712	7.55079	7.90748	7.733142
**U251 cells**								

	Lenti-Vector				Lenti-RASD1			
0h	1	1	1	1	1	1	1	1
24h	1.195686	1.427838	1.220339	1.275809	1.196411	1.479561	1.579262	1.473579
48h	1.865434	1.60452	1.442219	1.450437	1.870389	1.974078	2.087737	1.994018
72h	2.50642	2.263996	2.660503	2.568053	2.959123	2.534397	2.428714	2.564307
96h	2.664612	2.810478	2.861839	3.233693	2.402792	2.836491	2.698604	2.497707
120h	3.714432	3.827427	3.884951	3.989728	4.179461	4.227318	4.165504	4.325025
**U87 cells**								

rFigure 1. EdU assay assessed the proliferation in glioma cells

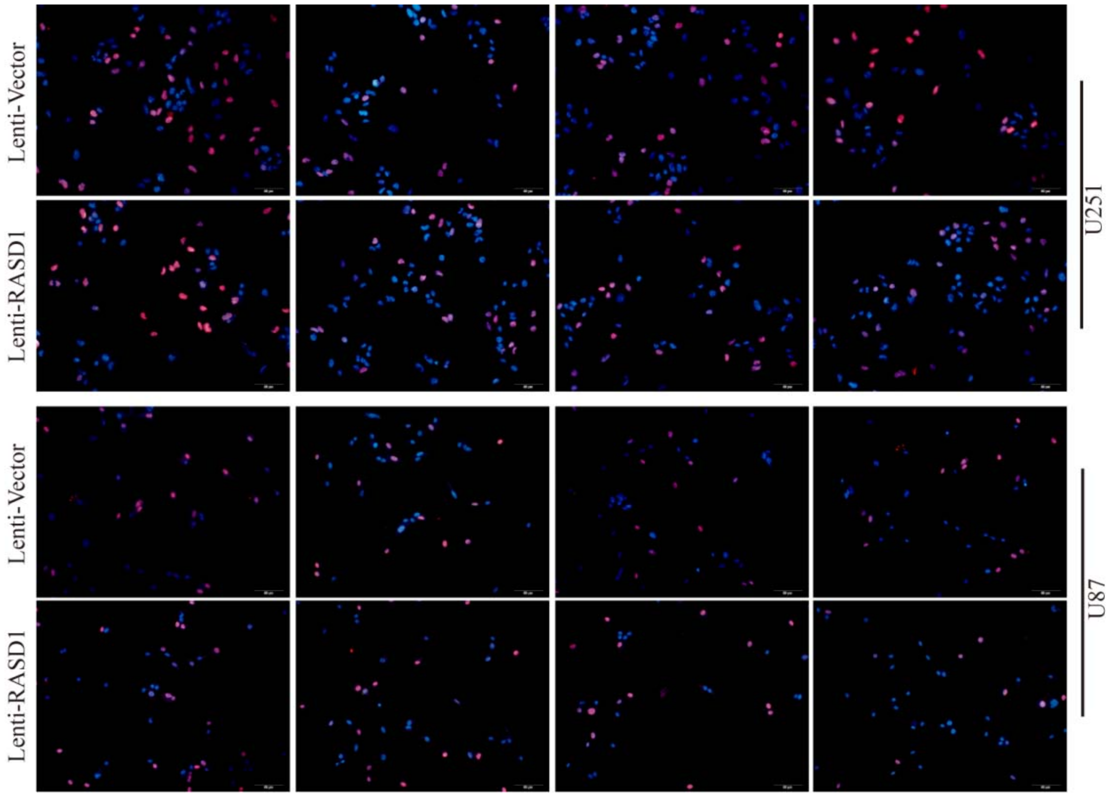

rFigure 2. Colony formation assay exhibited the proliferation in glioma cells

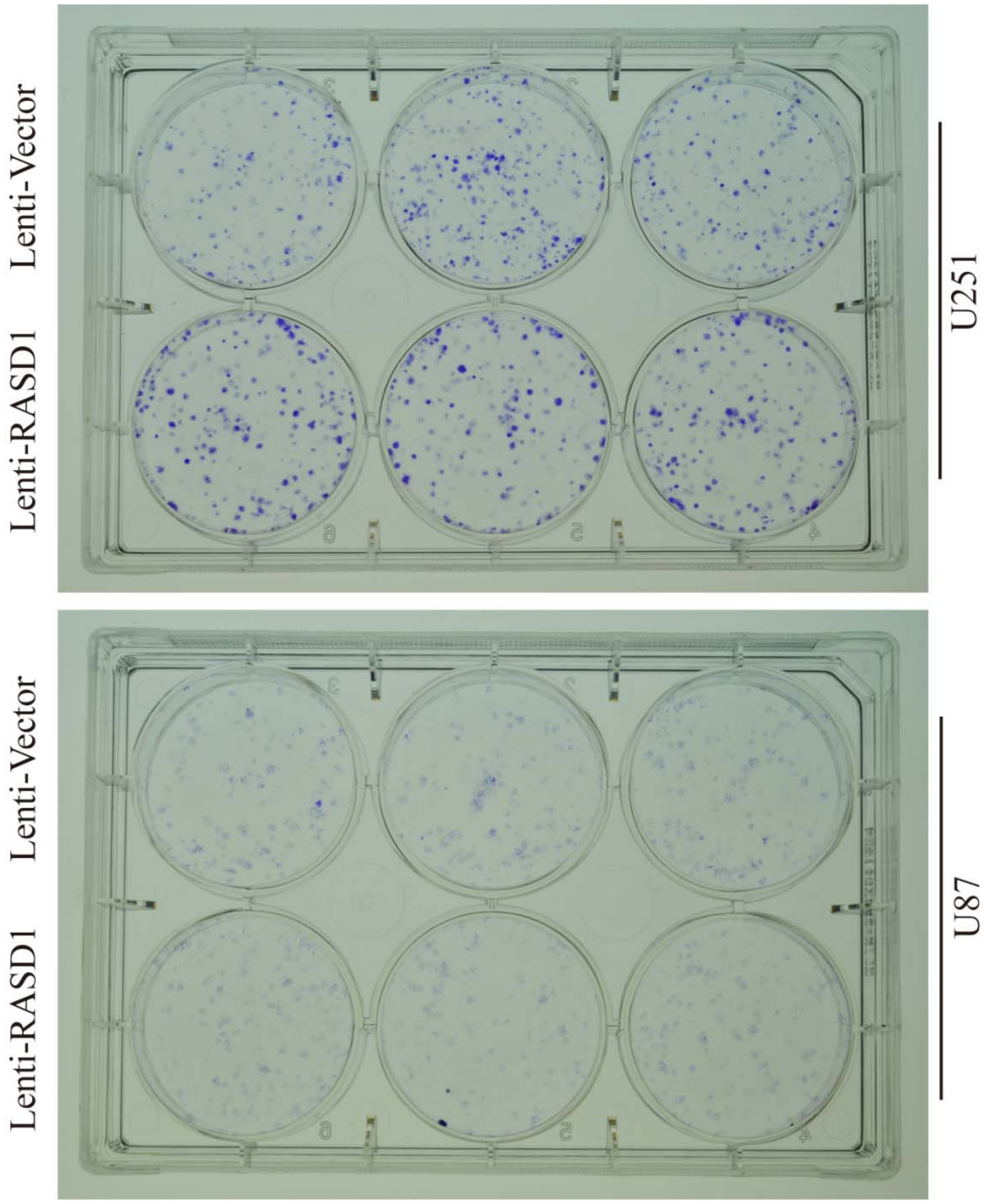

rFigure 3. Flow cytometry showed the cell cycle progression in glioma cells

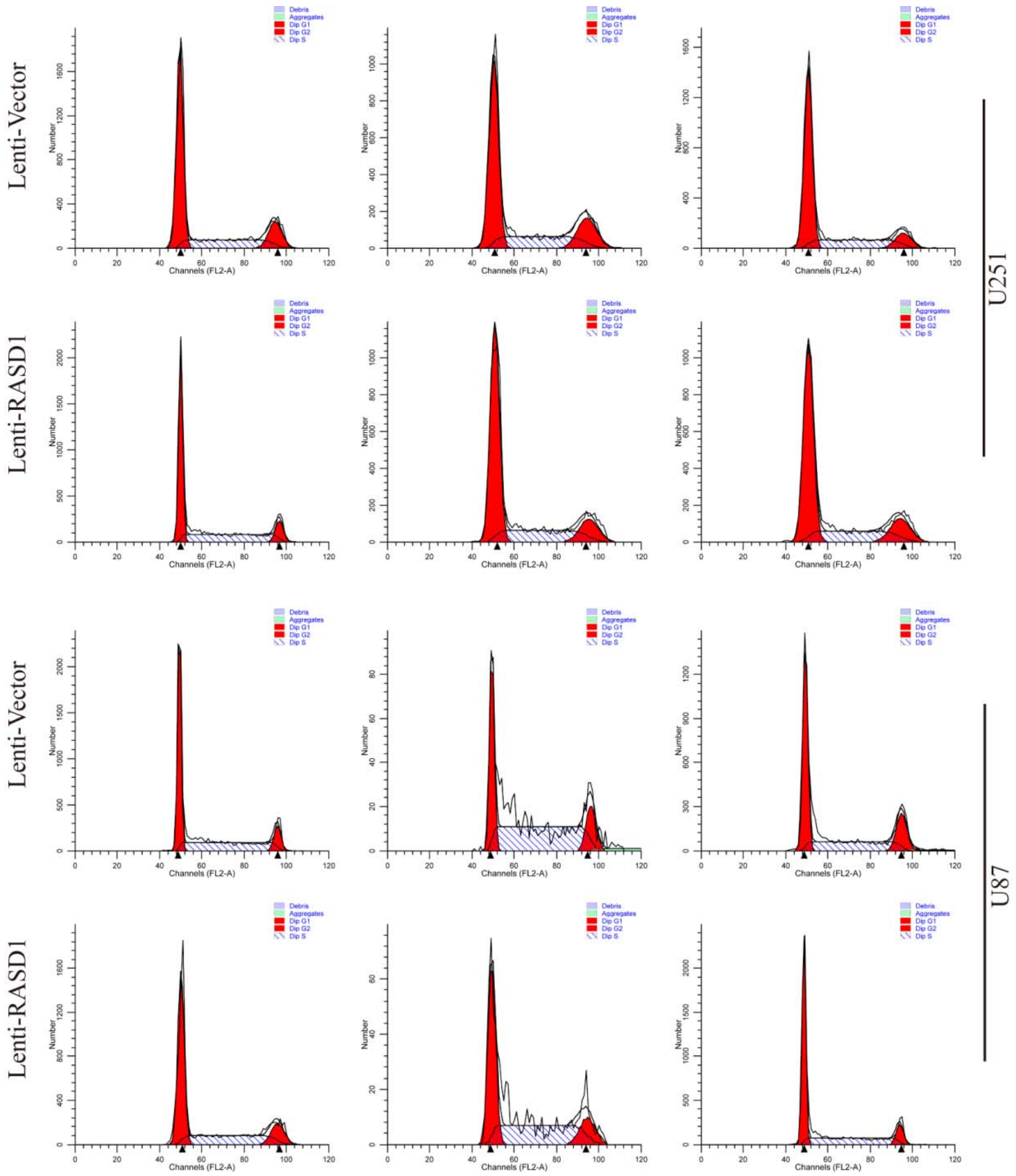

rFigure 4. Would healing assay showed the migration ability of glioma cells

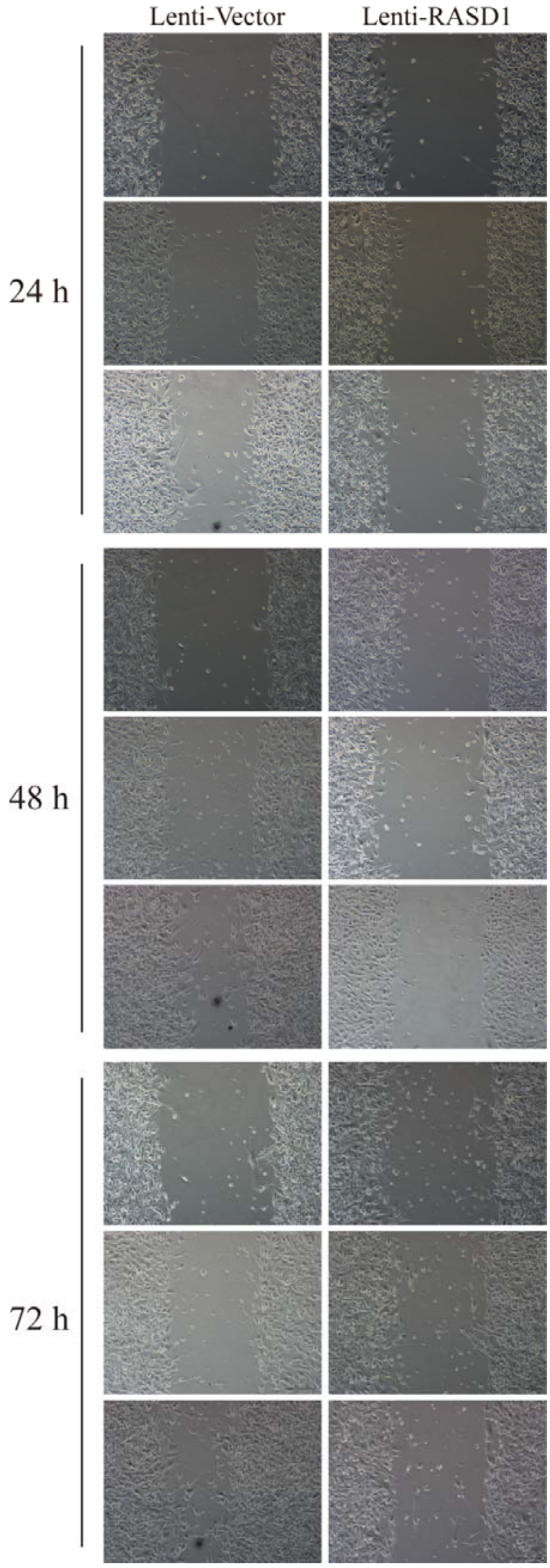

rFigure 5. Transwell migration assay exhibited the migration ability of glioma cells

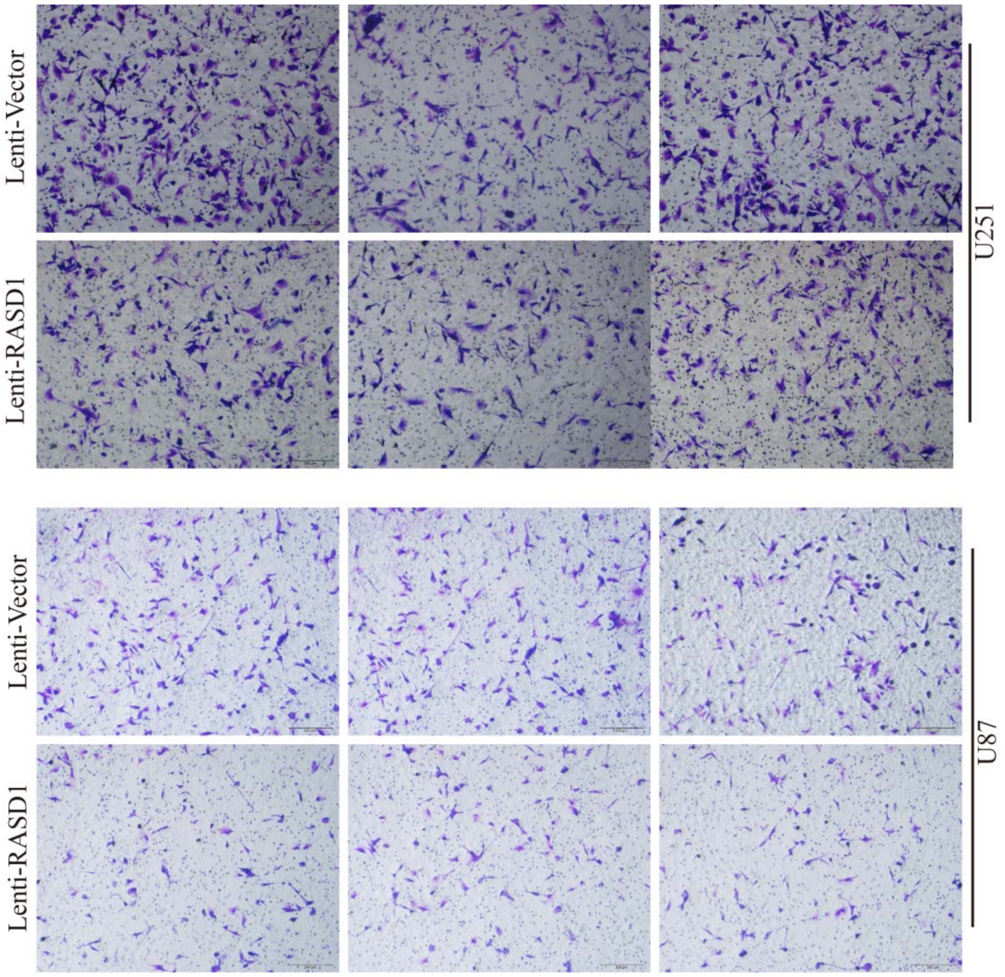

rFigure 6. Transwell invasion assay showed the invasion ability of glioma cells

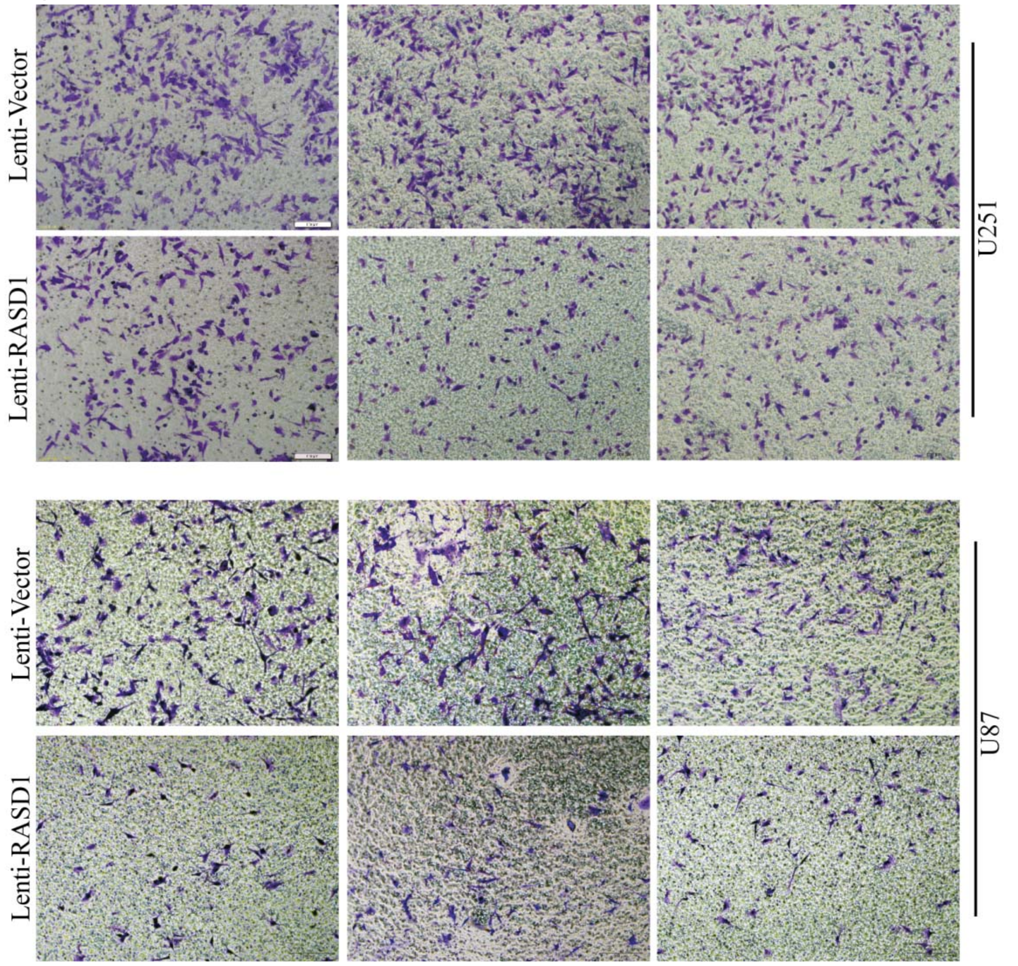

rFigure 7. Lower power images for transwell assay

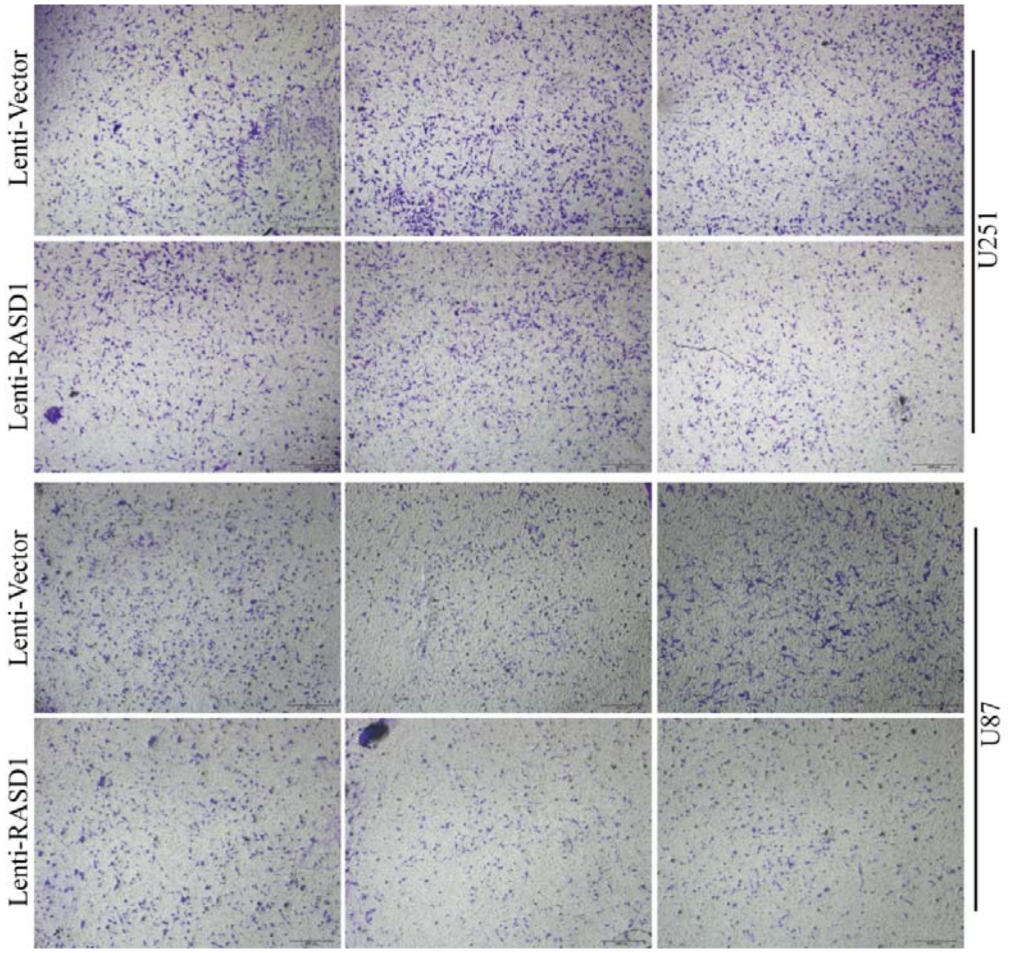

rFigure 8. MMP2 Western blot assessed the invasion ability in glioma cells

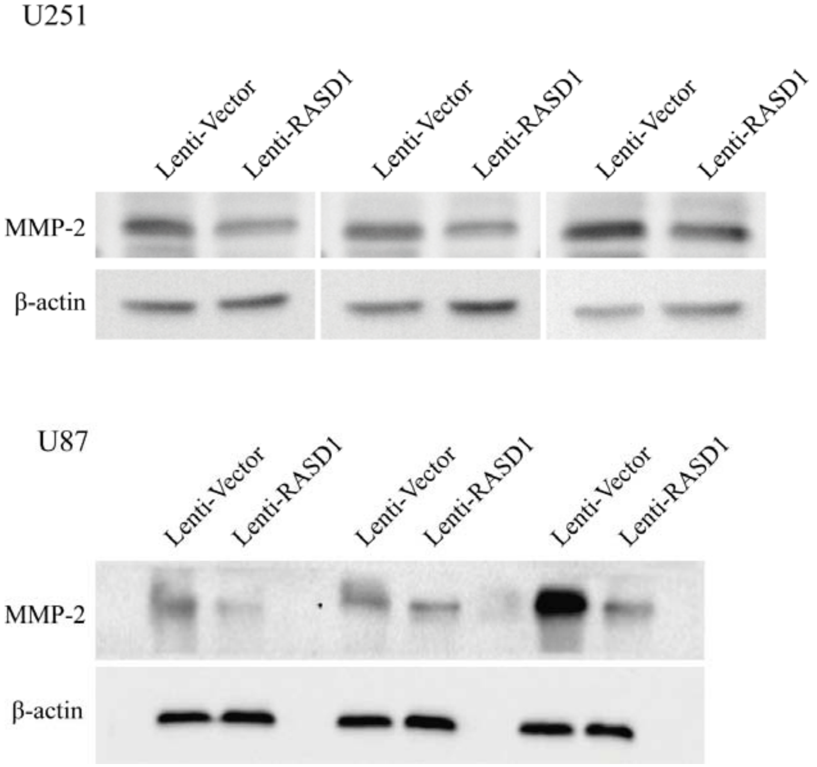

rFigure 9. Antibody array revealed the signaling pathways in glioma cells

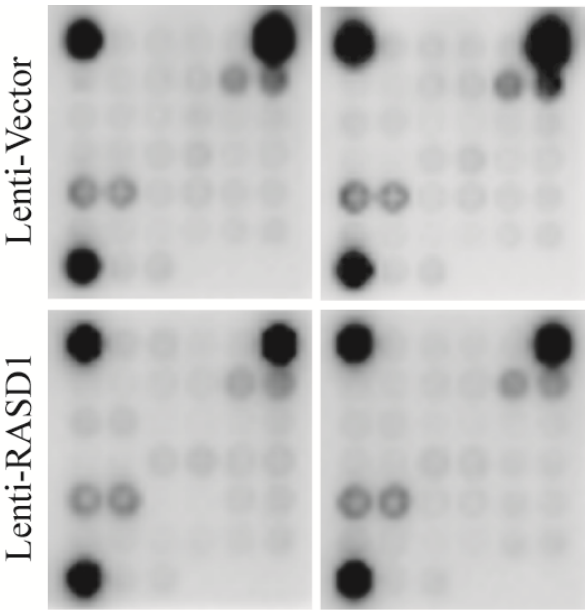

rFigure 10. Western blot detected the signaling molecules in glioma cells

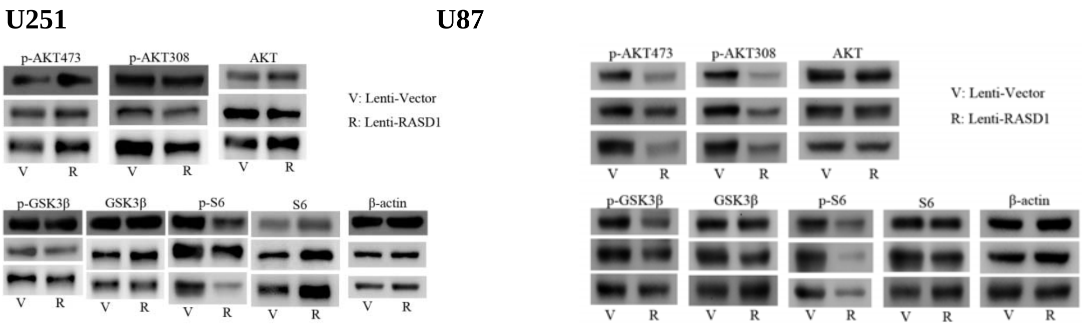

rFigure 11. Images for analysis of the tumor volume in the intracranial glioma xenograft model

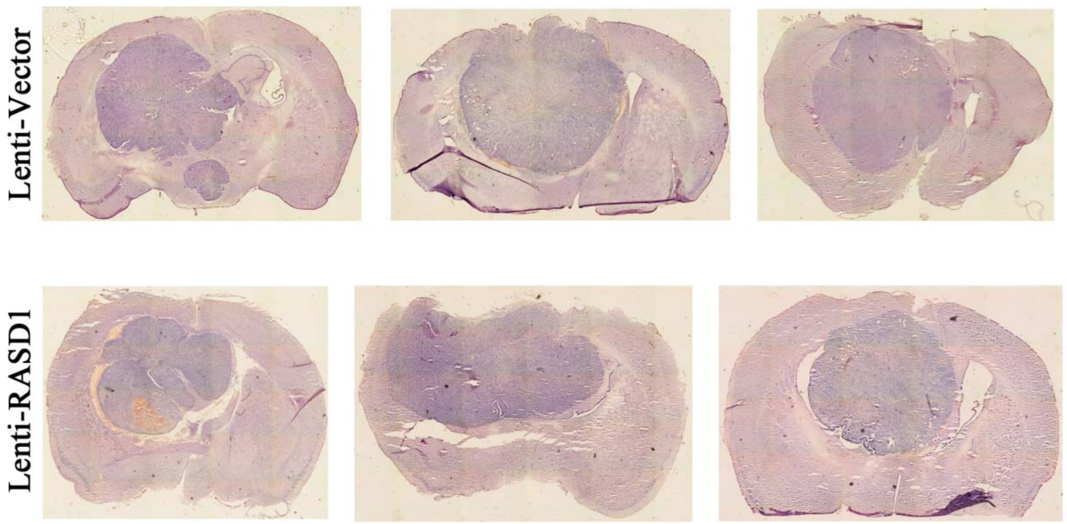

rFigure 12. Images for analysis of the invading cell numbers in the intracranial glioma xenograft model

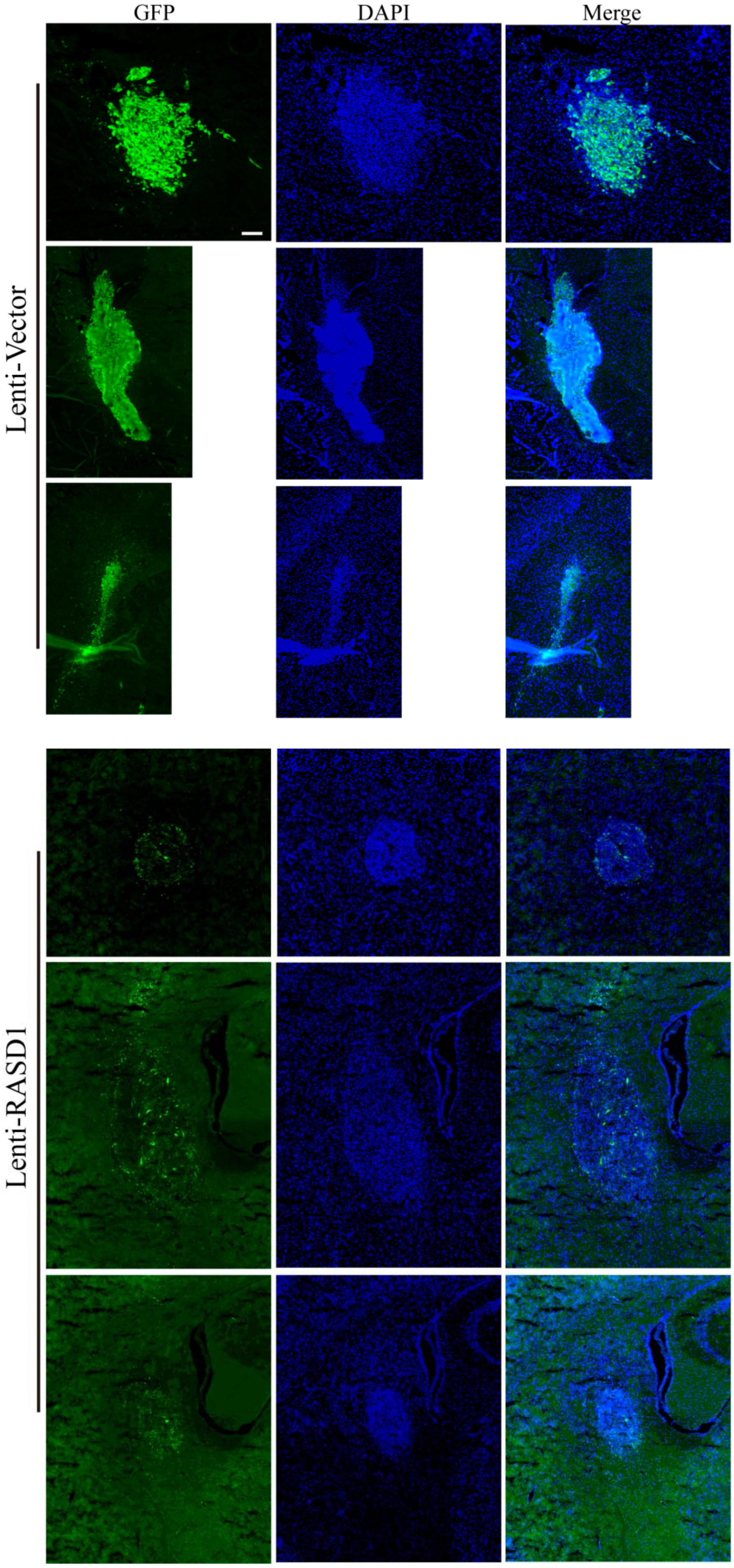

Supplement: Supplementary file 1 — Supplementary information [file 41598_2017_3612_MOESM1_ESM.pdf]
